# ﻿An integrated taxonomic revision of *Ctonoxylon* (Coleoptera, Curculionidae, Scolytinae) reveals new Malagasy species originating from multiple recent colonisations of the island

**DOI:** 10.3897/zookeys.1203.123757

**Published:** 2024-05-28

**Authors:** Bjarte H. Jordal

**Affiliations:** 1 Department of Natural History, University Museum of Bergen, University of Bergen, P.O. 7800, NO-5020 Bergen, Norway University of Bergen Bergen Norway

**Keywords:** Afrotropical, bark beetles, biogeography, Madagascar, taxonomy, Xyloctonini

## Abstract

*Ctonoxylon* is a strictly Afrotropical genus of bark beetles breeding under bark of rainforest trees and lianas. A taxonomic revision of the genus included a molecular phylogenetic analysis of ten species based on three gene fragments and was compared to a morphology-based tree topology for all 24 currently recognised species. Four species are described as new to science: *Ctonoxylontorquatum*, **sp. nov.**, *Ctonoxylontuberculatum*, **sp. nov.**, *Ctonoxylonquadrispinum*, **sp. nov.**, all from Madagascar, and *Ctonoxylonpilosum*, **sp. nov.** from Cameroon. *Ctonoxylonhirsutum* Hagedorn, 1910, **stat. rev**. is resurrected from synonymy with *C.flavescens* Hagedorn, 1910, and *C.atrum* Browne, 1965 **stat. rev.** from its synonymy with *C.methneri* Eggers, 1922 (as *C.hamatum* Schedl, 1941). The following species have new synonymies suggested: *Ctonoxylonfestivum* Schedl, 1941 (= *C.dentigerum* Schedl, 1941, **syn. nov.**), *C.methneri* Eggers, 1922 (= *C.hamatum* Schedl, 1941, **syn. nov.**, = *C.griseum* Schedl, 1941, **syn. nov.**), *C.montanum* Eggers, 1922 (= *C.longipilum* Eggers, 1935, **syn. nov.**, = *C.nodosum* Eggers, 1940, **syn. nov.**), *C.camerunum* Hagedorn, 1910 (= *C.conradti* Schedl, 1939, **syn. nov.**), and *C.spinifer* Eggers, 1920 (= *C.setifer* Eggers, 1920, **syn. nov.**). New country records are noted for *C.festivum* (Tanzania), *C.flavescens* (Uganda), *C.camerunum* (Liberia), *C.crenatum* Hagedorn, 1910 (Democratic Republic of the Congo), *C.spathifer* Schedl, 1941 (Ghana), *C.atrum* (Cameroon), and *C.spinifer* (Madagascar), with patterns in distribution and colonisation of Madagascar discussed. An identification key with pictures of all species is provided.

## ﻿Introduction

The Afrotropical genus *Ctonoxylon* Hagedorn, 1910 is a member of the bark beetle tribe Xyloctonini Eichhoff, 1878. Although some dubious records have been noted from Madagascar (Schedl 1977), the large majority of collections are from western parts of Africa and particularly the Congolese basin (Schedl 1956, 1957, 1961). Only two species are restricted to the eastern Zambezian and southern African parts of the Afrotropics and five primarily western species extend their distribution to the eastern tropical Africa (Table 1). As for most Afrotropical bark beetles, knowledge about this genus was previously limited to a few commonly collected species.

**Table 1.** Currently valid species of *Ctonoxylon* Hagedorn, and their validated distribution (DRC = Democratic Republic of the Congo).

*Ctonoxylonacuminatum* Schedl, 1957	Nigeria, DRC (Dem. Rep. Congo)
*Ctonoxylonamanicum* Hagedorn, 1912	Cameroon, Tanzania
*Ctonoxylonatrum* Browne, 1965	Cameroon, Nigeria
*Ctonoxylonauratum* Hagedorn, 1910	Cameroon, DRC
*Ctonoxylonbosqueiae* Schedl, 1962	Ghana
*Ctonoxyloncamerunum* Hagedorn, 1910	Ivory Coast, Ghana, Nigeria, Cameroon, DRC
*Ctonoxyloncaudatum* Schedl, 1971	DRC
*Ctonoxyloncornutum* Eggers,1943	Cameroon
*Ctonoxyloncrenatum* Hagedorn, 1910	Nigeria, Cameroon
*Ctonoxylonfestivum* Schedl, 1941	Cameroon, Eq. Guinea
*Ctonoxylonflavescens* Hagedorn, 1910	Tropical/subtropical Africa
*Ctonoxylonhirsutum* Hagedorn, 1910	Ghana, Cameroon
*Ctonoxylonhirtellum* Schedl, 1971	DRC
*Ctonoxylonkivuensis* Schedl, 1957	DRC
*Ctonoxylonmethneri* Eggers, 1922	Kenya, Tanzania, S. Africa
*Ctonoxylonmontanum* Eggers, 1922	Tropical Africa
*Ctonoxylonpilosum* Jordal, sp. nov.	Cameroon
*Ctonoxylonpygmaeum* Eggers, 1920	Cameroon
*Ctonoxylonquadrispinum* Jordal, sp. nov.	Madagascar
*Ctonoxylonspathifer* Schedl, 1951	Ivory Coast, Tanzania
*Ctonoxylonspinifer* Eggers, 1920	Tropical Africa, Madagascar
*Ctonoxylontorquatum* Jordal, sp. nov.	Madagascar
*Ctonoxylontuberculatum* Jordal, sp. nov.	Madagascar
*Ctonoxylonuniseriatum* Schedl, 1965	Namibia, S. Africa

*Ctonoxylon* is a peculiar group of species which are readily recognised by having divided eyes, a rounded pea-like body shape, impressions on lateral sclerites of the metathorax to accommodate the legs in resting position, and likewise, their tibiae have furrows for hiding the tarsi. As in the similar and related genera *Xyloctonus* Eichhoff, 1872, *Cryphalomimus* Eggers, 1927 and *Scolytomimus* Blandford, 1895, they have evident behavioural and morphological adaptations to avoid predators such as ants (Jordal 2024). The odd morphology inspired Eichhoff (1878), Hopkins (1915), and Schedl (1961) to elevate the tribe to subfamily or family status.

*Ctonoxylon* is the sister group to three other genera in subtribe Xyloctonina, based on molecular and morphological phylogenetic analyses (Jordal 2023, 2024), and is the most species rich. Approximately 29 species are listed in Wood and Bright (1992), with a few synonyms subsequently suggested or restated by Beaver (2011). Many more synonyms and new species are anticipated. Very few taxonomic revisions have been made over the last century, with mainly single species descriptions published and no keys were ever produced to enable proper identification of species. This study revises the genus, describes four species as new to science, and summarises known ecological and biological features of the genus. Three dozen new records are reported, many with new country or host plant records. An identification key is provided and is illustrated by photos of all species.

## ﻿Materials and methods

Type material and newly recorded samples were studied and deposited in the following collections:

**CAS** California Academy of Science, San Francisco, USA

**CMNC** Canadian Museum of Nature, Ottawa, Canada

**MNHN** Muséum National d’Histoire Naturelle, Paris, France

**MSUC** Michigan State University, AJ Cook arthropod research collection, East Lansing, USA

**NHMUK** The Natural History Museum, London, UK

**NHMW** Naturhistorisches Museum, Vienna, Austria

**RMCA** Musee Royal de l’Afrique Centrale, Tervuren, Belgium

**SDEI** Senckenberg Deutsches Entomologisches Institut, Muncheberg, Germany

**USNM** National Museum of Natural History, Washington D.C., USA

**ZFMK** Zoologisches Forschungsmuseum “Alexander Koenig”, Bonn, Germany

**ZMHB** Museum für Naturkunde der Humboldt-Universität, Berlin, Germany

**ZMUB** University Museum of Bergen, Norway

New specimens were collected during several field expeditions to Madagascar and to several African countries between 2006 and 2019. A few unidentified samples were collected by other researchers, in flight intercept or Malaise traps, or by light traps. Beetles collected by the author were dissected from dead woody materials, including lianas, seeds, twigs, branches, and tree trunks. Due to the distinct engravings by the beetles under bark, or in the wood, their family structure, brood size and stage was noted, and whether or not parents stayed with their progeny during their development.

**Figures 1–9.**
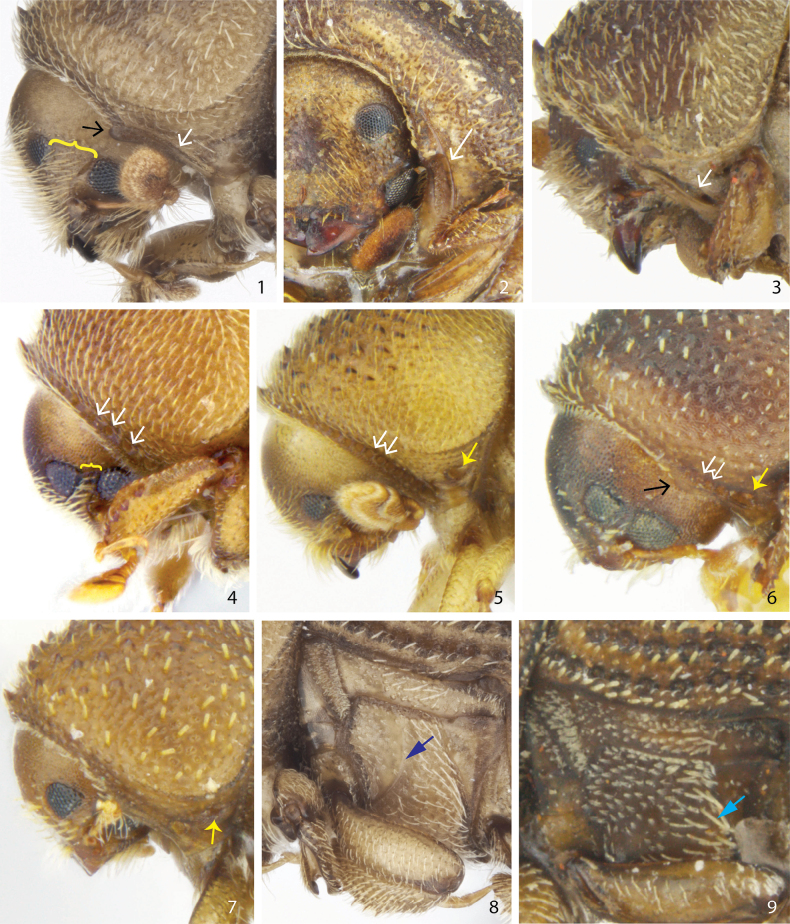
Novel characters in the identification of *Ctonoxylon* species. Curly brackets illustrate **1** widely separated eye parts and **4** a narrow separation by less than half the size of upper part. Black arrows indicate **1** a sharply pointed eye scraper or **6** a reduced and rounded nodule. White arrows indicate **1**–**2** the position of a sharp carina running from just above the eye scraper to procoxa, or in that same position **3** an elongated groove, or **4–6** a series of deep pits. Yellow arrows indicate **5–7** the position of a propleural pit. Dark blue arrow **8** points at the swollen mark from the mesotibia’s resting position. The pale blue arrow **9** indicates unusually broad and elongated setae on the posterior part of the metaventrite.

Morphological characters which are important for distinguishing species groups were included in a phylogenetic analysis (Tables 2, 3). Several of these characters are, despite their peculiar expression, new in taxonomic work on the genus. All *Ctonoxylon* species have divided eyes but the distance between the eyes varies much more than previously reported (Figs 1–6). Certain groups also have an eye scraper associated with the eye partition; it is shaped as a projection from the anterior lateral margin of the prothorax and fits in line with a tiny carina located between the two eye parts (Figs 1, 2). Another overlooked character includes a circular or slightly transverse groove just above the procoxa, reminiscent of a mycangium (Figs 5–7). It is not evident which purpose this groove may have, if any. Just above the procoxa, but in front of the just mentioned groove, a remarkable feature appears in some species in which they have a pitted collar running along the anterior margin of the prothorax (Figs 3–6). Other species have just a single vertically elongated pit (Fig. 7) or a longer groove parallel to the front margin (Fig. 3). Yet another group of species have in the same position a long vertical carina, replacing the groove or series of pits.

**Table 2.** Morphological characters coded for *Ctonoxylon* and hypothetical outgroup.

1. Eyes: 0, each eye part separated by little more than scapus thickness; 1, separated by width of upper eye or more.
2. Eye scraper on the prothorax margin: 0, absent; 1, a small, rounded nodule; 2, an acuminately shaped tooth.
3. Frons vestiture: 0, fine hair-like setae; 1, scale-like setae; 2, glabrous.
4. Anterior margin of the pronotum: 0, smooth; 1, with a single fused tooth; 2, with pair of subcontiguous teeth; 3, with four teeth.
5. Pronotal setae: 0, fine hair-like setae only; 1, scattered coarse setae, sometimes mixed with finer setae; 2, glabrous.
6. Just inside the anterior lateral margin of the prothorax: 0=smooth; 1, long carina from eye scraper to procoxa; 2, carina replaced by an elongated cavity; 3, replaced by a series of deep pits.
7. Propleuron, just above procoxa: 0, smooth; 1, with deep elongate or circular pit.
8. Main setae on the elytral interstriae: 0, hairlike; 1, scalelike; 2, absent.
9. Interstrial ground vestiture: 0, hair-like; 1, scale-like; 2, absent.
10. Elytral apex: 0, emarginate; 1, rounded; 2, pronged.
11. Setae on the posterior part of the metaventrite: 0, hairlike; 1, short and broad; 2, very long and broad.
12. Metaventrite: 0, smooth; 1, with a vertical curved swollen edge demarcating the posterior position of the mesotibia.
13. Elytral suture locking mechanism: 0, normal straight; 1, buckled suture at elytral midlength.
14. Protibial groove on its anterior face: 0, tiny or absent; 1, shallow, no more than half the width of protibia; 2, as deep as width of tibia.

**Table 3.** Data matrix based on coded character states from Table 2.

outgroup	0 0 0 0 0 0 0 0 0 0 0 0 0 0
*Ctonoxylonhirtellum*	0 0 0 2 0 1 0 0 2 0 0 1 0 2
*C.festivum*	1 2 0 2 0 1 0 0 0 0 0 1 0 2
*C.flavescens*	1 2 0 2 0 1 0 0 2 0 0 1 0 2
*C.tuberculatum*	1 2 0 2 0 1 0 0 2 0 0 1 0 2
*C.hirsutum*	1 2 0 2 0 1 0 0 0 0 0 1 0 2
*C.bosqueiae*	1 2 0 2 0 1 0 0 2 0 0 1 0 2
*C.montanum*	1 2 0 2 0 1 0 0 2 2 0 1 0 2
*C.cornutum*	1 2 0 2 0 1 0 1 1 0 1 1 1 2
*C.camerunum*	1 2 0 2 0 1 0 1 1 0 1 1 1 2
*C.torquatum*	0 0 0 2 0 3 1 1 1 2 0 0 0 1
*C.pilosum*	0 0 0 2 0 3 1 1 0 0 0 0 0 1
*C.auratum*	0 0 1 1 0 3 1 1 0 0 0 0 0 1
*C.caudatum*	1 1 2 2 2 2 1 1 1 2 0 0 0 1
*C.pygmaeum*	0 1 2 2 1 3 1 1 2 2 0 0 1 2
*C.crenatum*	1 1 0 2 2 2 1 2 2 2 0 0 0 ?
*C.kivuensis*	1 1 0 2 0 2 1 0 2 1 0 0 0 ?
*C.spathifer*	1 0 0 1 0 2 1 1 1 1 0 0 0 1
*C.quadrispinum*	1 1 0 4 0 2 1 1 1 0 0 0 1 2
*C.methneri*	1 1 0 2 0 2 1 1 1 0 1 0 1 2
*C.atrum*	1 1 0 2 0 2 1 1 1 0 1 0 1 2
*C.acuminatum*	1 1 0 2 0 2 1 1 2 1 0 0 1 2
*C.amanicum*	0 0 1 2 1 2 1 1 2 1 2 0 1 2
*C.spinifer*	1 1 1 2 1 2 1 1 2 1 2 0 1 2
*C.uniseriatum*	1 0 1 2 1 2 1 1 2 1 2 0 1 2

Phylogenetic analyses of molecular and morphological data were executed in MrBayes. Molecular data from the three gene fragments mitochondrial cytochrome oxidase 1 (COI), elongation factor 1 alpha (EF-1a), and the large ribosomal subunit (28S), were previously analysed and reported in Jordal (2023). New morphological data were analysed with MrBayes using 5 million generations of four chains run in parallel with one cold chain of temp=0.3; character variation followed a gamma distribution of variable rates. These data were also analysed by parsimony in PAUP* using implied weighting (e.g., in Goloboff et al. 2018) to increase resolution in the tree topology.

Biogeographical inference is based on a recently published study (Jordal 2024) on the related genus *Xyloctonus*. The definition of ancestral areas is based on a statistical clustering algorithm for plants and four animal groups (Linder et al. 2012). This classification is very similar to more traditional classifications (e.g. Morrone and Ebach 2022) but is firmly founded on statistical similarity in fauna and flora.

## ﻿Results and discussion

### ﻿Phylogeny and biogeography

**Figures 10–11.**
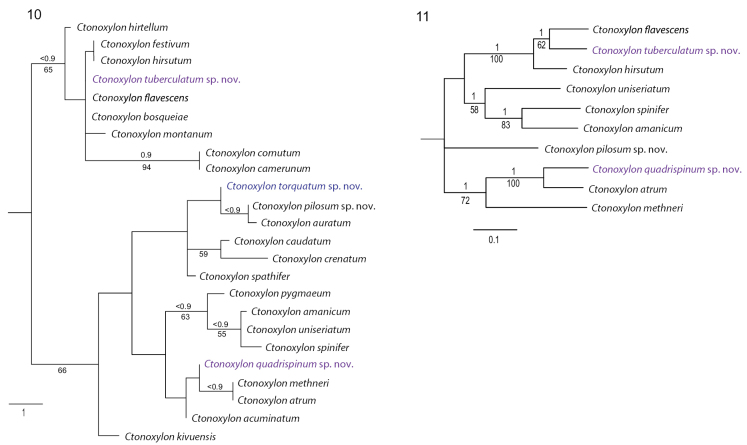
Phylogeny of *Ctonoxylon*. Node support is given as posterior probabilities above and parsimony bootstrap values below nodes **10** tree topology resulting from the parsimony analysis of 14 morphological characters for all species using implied weighting (Goloboff et al. 2018) **11** partial tree topology redrawn from a previously published Bayesian tree topology based on 1958 nucleotide position from three gene fragments (Jordal 2023). Species found in Madagascar marked in purple.

Bayesian and parsimony analyses of 14 morphological characters coded for all valid species in the genus resulted in a poorly resolved tree topology. Using implied weights, the parsimony tree was more fully resolved (Fig. 10). A dichotomy appeared between a group of taxa related to *C.flavescens*, and taxa related to *C.methneri*, respectively. Species in these two clades differ consistently by the presence of a carina along the anterior lateral margin of the prothorax as seen in the *flavescens* group (versus grooves or deep pits), in the absence of a propleural pit above the procoxae as seen in all species in the *methneri* clade, and the demarcation of the mesotibiae in resting position in the *flavescens* clade, which imprints a glabrous and swollen area on the anterior half of the metaventrite and metanepisternum. Species in the *flavescens* group, with one exception, have a sharp eye scraper pointing from the anterior lateral margin of the prothorax, in line with a fine horizontal carina between the two eye parts (Fig. 1).

Bayesian analysis of nucleotides from three gene fragments partially supported the two main groups described above (Fig. 11). A major difference from the morphological analyses was the grouping of *C.uniseriatum*, *C.spinifer*, and *C.amanicum* as sister to the *flavescens* group, albeit without any strong node support. The separation of *C.pilosum* sp. nov. and a group consisting of *C.methneri*, *C.atrum*, and *C.quadrispinum* is also consistent with morphology. It is furthermore notable that *C.atrum* separates from *C.methneri*, with which it was previously synonymised, and instead forms a sister relationship with *C.quadrispinum* sp. nov., documenting considerable genetic divergence between the three species. A similar degree of genetic divergence was found between the morphologically very similar species in the *flavescens* clade (Table 4), and in the *spinifer* clade (Fig. 11). These data suggest that rather minor morphological variation warrants studies on genetics to test species validity and potential cryptic speciation.

**Table 4.** Genetic distances for species in the *C.flavescens* complex. COI p-distances in the lower left triangle, and 28S p-distances in upper right. The two samples of *C.flavescens* are from Uganda (U) and Cameroon (C).

		28S			
		*flavescens* - U	*flavescens* - C	*hirsutum*	*tuberculatum*
**COI**	*flavescens* - U	–	0.0	2.6	1.8
	*flavescens* - C	10.3	–	2.6	1.8
	*hirsutum*	16.3	15.4	–	1.0
	*tuberculatum*	13.2	12.2	14.9	–

Recent biogeographical and phylogenetic analysis of Xyloctonini (Jordal 2023, 2024), including the morphology-based analyses in this study (Fig. 10), revealed three putatively recent origins of undescribed species in Madagascar. Reconstruction of ancestral areas using molecular data for two of these species strongly supported a Congolese distribution of their ancestors. This is also the most likely hypothesis for the third species, *C.torquatum* sp. nov., for which DNA data was not possible to obtain. Based on morphological analysis, this species is closely related to the two Congolese species *C.auratum* and *C.pilosum* sp. nov., strongly suggesting an origin in that area.

### ﻿Biology

All species of *Ctonoxylon* observed in the field during this study established monogynous pairs under bark. The female first initiated a tunnel opening and thereafter let one of the many wandering males mates with her, in or near the entrance. The male stayed with the female for some time during which the female engraved a tunnel where eggs were laid in designated pits along the tunnel wall. In two species the tunnel was made longitudinally to the wood grain, whereas in three other species they cut tunnels transversely to the grain (Table 5). Brood sizes ranged from fewer than 10 eggs up to more than 50 and did not generally correlate with host plant diameter, except always low for *C.uniseriatum* breeding in very thin lianas. Males left the tunnel system early in all observed species, usually before the first eggs hatched, otherwise at early larval stage. The female also left her offspring before they were completely developed, approximately at late larval or pupal stage.

**Table 5.** Reproductive biology observed for *Ctonoxylon* species (*records from Schedl 1961). All observed species form monogynous pairs at the early stage of mating and egg laying. The two last columns indicate the offspring developmental stage where parents have permanently left the nest.

Species	Host family (majority)	Diam (cm)	egg tunnel direction	Brood size	males leave	females leave
Ctonoxylonacuminatum*	Apocynaceae	5	transverse	36		
*Ctonoxylonamanicum*	(liana)	0.6–2.0	irregular			
*Ctonoxylonflavescens*	Moraceae	15	longitudinal	19-23	egg	pupa
	Malvaceae	7	longitudinal	20-30	larva	
	Apocynaceae*	2–4	longitudinal	30-45		
*Ctonoxylonmethneri*	Oleaceae	2–60	transverse	8–48	egg	larva
*Ctonoxylonquadrispinum*	(liana)	4	transverse	50–60		pupa?
*Ctonoxylonuniseriatum*	(liana)	0.8–3	longitudinal	3–8	egg	larva

Host plants were rarely identified in past studies, but host selection appears to be broad in *C.flavescens*, and in this study always found in branches of fallen trees. One of the host plant families previously recorded for this species, Apocynaceae, was also recorded for *C.acuminatum* (see Schedl 1961), which may indicate some generality across species. It should also be noted that *C.flavescens* has been recorded most frequently from fig trees, indicating a likely important host plant. Similarly, *C.methneri* was in this study repeatedly collected from cape olive trees (*Oleacapensis* L.), where they often breed in huge trunks with very thick bark. Yet other species, such as *C.uniseriatum*, *C.amanicum* and *C.quadrispinum*, were found only in thin lianas, demonstrating huge variability in host preferences across the genus.

### ﻿Taxonomy

#### ﻿Genus Ctonoxylon

Taxon classificationAnimaliaColeopteraCurculionidae

Hagedorn, 1910

AEB09E4C-1DDA-5890-93CC-674438501581

##### Type species.

*Ctonoxylonauratum* Hagedorn, 1910: 4, subsequent designation by Hopkins (1914): 119.

##### Diagnosis.

Typical for subtribe Xyloctonina, with stout and rounded body shape, eyes divided, and protibia on its anterior face with a deep groove. Antennal funiculus 7-segmented, club with oblique lateral septum, sutures faint or obscure, asymmetrical; pronotum with pair of teeth in females at the anterior margin, in males just behind anterior margin, teeth occasionally fused, or divided into four parts. Elytral declivity steep, abdominal ventrites flat or gently rising towards elytral apex.

##### Sexual dimorphism.

Dimorphism between males and females has not been clearly formulated in previous work. Nevertheless, the male pronotum has a pair of raised teeth located a little behind the front margin whereas the females have the pair of teeth at the margin and slightly closer to each other. Occasionally the male frons is also slightly modified in some species, either with the central area shinier, or with longer setae, or with patterns of transverse wrinkles. In at least one species (*C.montanum*) the degree of inflation of the elytral apex differs between the sexes (Schedl 1972).

##### Comments.

Recent phylogenetic analyses (Jordal 2023) supported a separate position of *Ctonoxylon* in Xyloctonina as the sister lineage to the three other genera *Scolytomimus*, *Xyloctonus*, and *Cryphalomimus*.

#### ﻿ Ctonoxylon hirtellum

Taxon classificationAnimaliaColeopteraCurculionidae

Schedl

AAD3E5AC-6D45-567B-A469-360DBA04B81C

Figs 12 , 15 , 18

Ctonoxylon hirtellum Schedl, 1971: 9.

##### Type material.

***Holotype***, male: Congo Belge [Democratic Republic of the Congo], Yangambi, 2.VII.1952, K.E. Schedl leg. [NHMW].

##### Diagnosis.

Length 1.5 mm, 2.1× as long as wide, colour pale brown. Eye parts closely separated (by scapus thickness); pronotal eye scraper weakly developed, faint carina near anterior lateral margin from scraper to coxa, without associated groove or propleural pit; scutellar shield at same level as elytra; elytral striae impressed, punctures slightly elongated, separated by 2–3× their diameter; elytral vestiture consisting of long hairlike setae separated within rows by a little less than their length; elytral apex slightly extended with sharp tubercles along the posterior margin.

##### Distribution.

Democratic Republic of the Congo.

##### Biology.

Nothing known except collected in a tropical lowland rainforest.

#### ﻿ Ctonoxylon festivum

Taxon classificationAnimaliaColeopteraCurculionidae

Schedl

9409D707-6C8A-5C2D-B228-7171B27904B9

Figs 13 , 14 , 16 , 17 , 19 , 20

Ctonoxylon festivum Schedl, 1941: 389.
Ctonoxylon dentigerum Schedl, 1941: 388, syn. nov.

##### Type material.

***Holotype***, female: Kamerun, Soppo, 800 m, XII 1912, v. Rothkirch S.G. Holotype of *C.dentigerum*, male: Spanish Guinea [Equatorial Guinea]. [both in NHMW].

##### Diagnosis.

Length 3.1–3.2 mm. 2.1–2.2× as long as broad; colour brown. Upper and lower eye parts separated by more than width of upper part; pronotal eye scraper acutely pointed; a sharp carina running from scraper to procoxa, without associated groove or propleural pit; scutellar shield at level with elytra; striae slightly impressed; main interstrial setae and ground vestiture similar and evenly distributed, each seta a little shorter than width of an interstriae; elytral apex slightly emarginated; resting position of mesotibiae marked on metaventrite.

##### Distribution.

Cameroon, Equatorial Guinea, Tanzania (new country record).

##### New record.

Tanzania, Morogoro, Kimboza Forest Reserve [GIS: -7.023, 37.806], S.S. Madoffe, leg. [1, NHMUK].

##### Comments.

Using the principle of first reviser the name *festivum* is given priority as there is nothing particularly dentigerous about this species. Differences noted between the holotypes of *C.dentigerum* and *C.festivum* are likely due to sexual dimorphism, with the male *dentigerum* having the anterior pair of teeth on pronotum a little behind the anterior margin and in *festivum* along the front margin. Minor variation in the length of elytral setae can also be due to dimorphism, or simply due to variation between individuals as often observed in similar species. Biology unknown, except collected in low- to mid-altitude rainforest.

**Figures 12–20.**
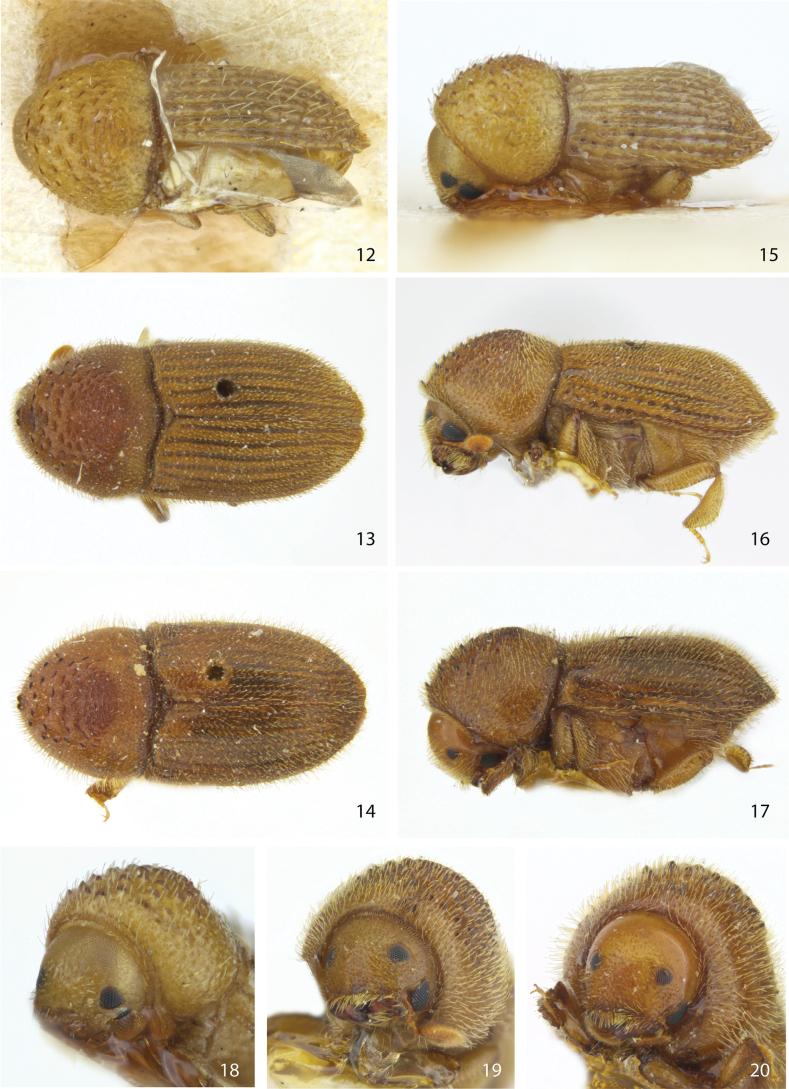
Dorsal, lateral, and front views of **12, 15, 18***Ctonoxylonhirtellum* male holotype **13, 16, 19***Ctonoxylonfestivum* female holotype, and **14, 17, 20***Ctonoxylondentigerum* male holotype, syn. nov. (= *Ctonoxylonfestivum*).

#### ﻿ Ctonoxylon flavescens

Taxon classificationAnimaliaColeopteraCurculionidae

Hagedorn

9C236C4C-9C63-5202-BADB-40E52A4580DF

Figs 21 , 24 , 27

Ctonoxylon flavescens Hagedorn, 1910: 4.
Ctonoxylon flavescens usambaricum Eggers, 1920: 38.
Ctonoxylon flavescens opacum Strohmeyer, nom. dub. – not published.

##### Type material.

***Holotype***: Kamerun [ZMHB]; ‘type’ of *C.flavescensopacum*: Kamerun [SDEI]. Holotype of *C.flavescensusambaricum*: Mkulumusiberg 1000 m, bei Sigi Ostafrika [NHMW].

##### Diagnosis.

Length 2.2–3.1 mm. 2.1–2.3× as long as broad; colour brown, dull. Upper and lower eye parts separated by more than width of upper part; pronotal eye scraper acutely pointed; a sharp carina running from scraper to procoxa, without associated groove or propleural pit; scutellar shield at level with elytra; striae distinctly impressed; interstrial setae bristle-like, variable in length and placed irregularly in partly confused rows, without ground vestiture; elytral apex slightly emarginated; resting position of mesotibiae marked on metaventrite.

##### Distribution.

Guinea, Ghana, Cameroon, Democratic Republic of the Congo, Gabon, Uganda (new country record), Tanzania.

##### New records.

Uganda, Masindi, Budongo, Nyabyeya [GIS: 1.673, 31.540], 3. July. 1998, *ex Ficus* branch, B. Jordal, leg. [ZMUB]; Budongo, BFP Station, Sonso [1.723, 31.545], 6.10.2004, T. Wagner leg. [1, ZFMK]; Kichwamba [0.71, 30.20], 25.04.1968, P.J. Spangler [1, USNM]; Cameroon, Limbe, Ekande [GIS: 4.081, 9.172], 1000 m. alt., 20. Nov. 2007, *ex Colaacuminata* standing tree, B. Jordal, leg [ZMUB]; [Ghana], ‘Gold Coast’, Takoradi [4.90, -1.75], 10.12.1946, ex bark of mahogany logs [*Khayaivorensis*] [4, USNM].

##### Biology.

Very little is known about this species despite frequent collections from many African countries. This study reports *Cola* as a new host plant genus in the same family Malvaceae as for the previously recorded *Triplochiton* (see Schedl 1961). Another new record from *Ficus* is in line with some other collections of closely related species taken from various Moraceae genera (see below). It is also reported here from African mahogany logs (Meliaceae), demonstrating a rather broad assembly of host plants. Records are generally from the bark of larger branches and trunks where the maternal egg tunnel is cut longitudinally. The male may stay at least until eggs are hatched, but not much longer (Table 5). Brood size is moderately large, with 19–45 eggs or larvae.

##### Comments.

This species and the next three are morphologically very similar and can easily be confused. DNA sequence data for COI and 28S from three of the species nevertheless clearly separate them (Table 4, Fig. 11). Eastern and western populations of *C.flavescens* are also deeply, albeit less, diverged in the mitochondrial COI gene, but, more importantly, identical at the nuclear 28S gene. It is advisable to apply DNA sequence data to identify species in this complex group. The record from Madagascar is likely confused with the new species *C.tuberculatum* described in this work.

#### ﻿ Ctonoxylon bosqueiae

Taxon classificationAnimaliaColeopteraCurculionidae

Schedl

7302EBD2-8A02-505E-B3A2-0E94DD628C91

Figs 22 , 25 , 28

Ctonoxylon bosqueiae Schedl, 1962: 66.

##### Type material.

***Holotype*** and additional non-types from the type locality of *C.bosqueiae*: Ghana, Bobiri, Kumasi [NHMUK].

##### Diagnosis.

Body length 2.2–2.5 mm, 2.2–2.3× as long as broad; colour dark brown, dull. Upper and lower eye parts separated by more than width of upper part; pronotal eye scraper acutely pointed; a sharp carina running from scraper to procoxa, without associated groove or propleural pit; scutellar shield at level with elytra; striae distinctly impressed; interstrial setae bristle-like, variable in length and placed irregularly in rows, without ground vestiture; elytral apex entire; resting position of mesotibiae marked on metaventrite.

##### Distribution.

Ghana.

##### Comments.

This species is very similar to *C.flavescens* but differs by the nearly closed gap between the apical tip of each elytron. Genetic data are needed to test the validity of this species. It is only known from the type locality in Ghana. Other published records are removed on the suspicion being the similar and commonly occurring *C.flavescens* or *C.hirsutum*. The hostplant *Trilepisium* is in the plant family Moraceae, similar to many other host records for the *flavescens* group.

#### ﻿ Ctonoxylon hirsutum

Taxon classificationAnimaliaColeopteraCurculionidae

Hagedorn stat. rev.

B0D4CA0E-BC35-55C0-B267-5E466C7632BA

Figs 23 , 26 , 29

Ctonoxylon camerunum hirsutum Hagedorn, 1910: 4.
Ctonoxylon flavescens hirsutum Hagedorn, transfer by Eggers 1920 and Schedl 1961.

##### Type material.

***Syntype*** (metatype *sensu* Eggers): Kamerun, Conradt leg. [NHMW].

##### Diagnosis.

Body length 2.5–3.0 mm, 2.2–2.3× as long as broad; colour pale brown. Male frons slightly flattened on lower half, very slightly shrivelled, surface finely rugose above, female frons smooth, vestiture in the frons of both sexes consisting of fine setae. Eye parts separated by the size of upper eye; antennal club sutures not clearly marked; anterior lateral margin of prothorax at middle with acute eye scraper; a sharp carina running from eye scraper to near procoxa; propleural pit absent; elytral vestiture on each interstriae consisting of two irregular rows of slightly curved bristle-like setae and scattered very fine hair-like setae; elytral suture straight; elytral apex slightly emarginated, a few small and sharp tubercles along the posterior margin. Metanepisternum with mixed short plumose and hair-like setae; metaventrite with simple hair-like setae; these sclerites on its anterior third glabrous, with a vertically curved swollen trace of mesotibia’s resting position.

##### Distribution.

Ghana, Cameroon, Democratic Republic of the Congo, Gabon.

##### New records.

Cameroon, Ekande, 5 km N Limbe [GIS: 4.081, 9.172], 1100 m alt., ex unknown liana, 18.XI.2007, A. Breistøl, leg. [1, ZMUB]; Cameroon, Mbalmayo Forest Reserve, Eboufek [GIS: 3.499, 11.883], 28.07.1993, FIT [1, NHMUK]; Republic of the Congo, 40–60 km ESE of Bomassa [2.04, 16.59], 18.04.1993, D.H. Chadwick leg. [1, USNM].

**Figures 21–29.**
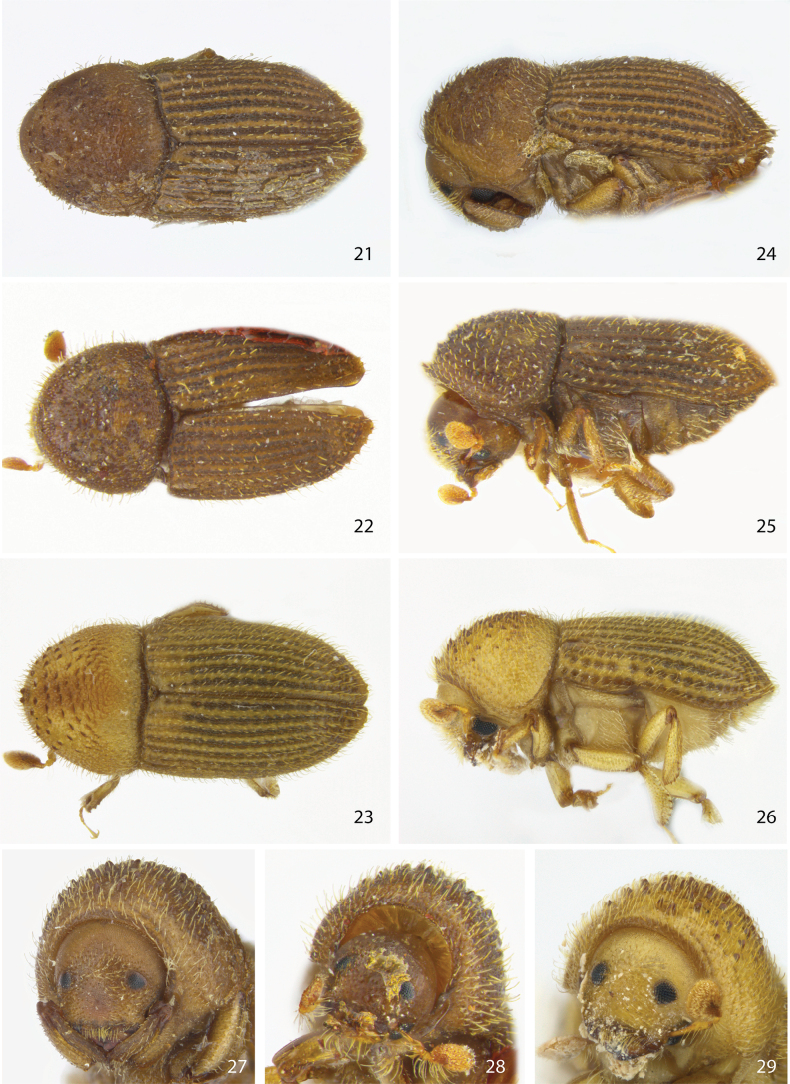
Dorsal, lateral, and front views of male **21, 24, 27***Ctonoxylonflavescens***22, 25, 28***Ctonoxylonbosqueiae* holotype, and **23, 26, 29***Ctonoxylonhirsutum* stat. rev.

##### Biology.

All records are from lowland rainforest sites in the western parts of Africa. It was previously collected from latex rich lianas (Schedl 1961). In the current study two further specimens were dissected from a dead liana together with specimens of *C.pilosum* sp. nov. One specimen was also collected in a flight intercept trap [NHMUK].

##### Comments.

Very similar to *C.flavescens*, except the elytral interstriae have fine pubescent ground vestiture and the two teeth near the anterior margin of the pronotum are subcontiguous. Previously designated as a variety of *C.flavescens*, but with a type designated, a subspecies name given and published by Hagedorn (1910). Sufficient diagnostic features as detailed by Eggers (1920), and molecular data demonstrating deep divergence from *C.flavescens* (Table 4), strongly support species status for the name *hirsutum*. Records from Ghana and Gabon (see Schedl 1961) could not be verified but these seem likely given several validated records from nearby countries.

#### ﻿ Ctonoxylon tuberculatum sp. nov.

Taxon classificationAnimaliaColeopteraCurculionidae

E94738BF-8DCA-5D94-B01E-4A87085E3EF4

https://zoobank.org/0921A825-BD54-4309-90AD-2345527F0034

Figs 30 , 33 , 36

##### Type material.

***Holotype***, male: Madagascar, Diana prov., Montagne d’Ambre [GIS: -12.54, 49.17], 1000 m alt., ex *Ficus* branch, 03.11.2019, B. Jordal, leg. [ZMUB]. Allotype [ZMUB] and paratype [NHMW]: same data as holotype.

##### Diagnosis.

Two teeth along the anterior margin of pronotum separated by more than width of a tooth; scattered interstrial setae separated within uniseriate rows by more than their length; posterior margin of elytra and declivital interstriae with sharp tubercles, largest tubercles on interstriae 1 and 3.

##### Description.

**Male.** Body length 3.0–3.1 mm, 2.1–2.2× as long as broad; colour brown. ***Frons*** flattened from upper level of eyes to epistoma, surface shrivelled; vestiture of fine short setae. Eyes divided, each part separated by a little more than size of upper half. Antennal funiculus 7-segmented; club setose, sutures and septum barely indicated. ***Pronotum*** coarsely asperate on anterior three quarters, two front teeth separated by little more than width of a tooth, located behind the margin. Anterior lateral margin of prothorax at middle with prominent eye scraper; a sharp carina running from eye scraper to near procoxa; propleural pit absent. ***Scutellar shield*** wider than long, oval. ***Elytral*** striae impressed, punctures irregular and small; interstriae rounded, densely micropunctate, vestiture consisting of uniseriate rows of slightly curved bristle-like setae and fine dense ground vestiture along the suture; elytral suture straight; elytral apex slightly emarginated, along the posterior margin and at each declivital interstriae with small sharp tubercles, at interstriae 1 and 3 tubercles 2–3× larger. ***Metanepisternum*** with mixed short bifid and longer bristle-like setae; metaventrite with simple hair-like setae; these sclerites on its anterior third partly glabrous and with small bifid setae, and with a swollen trace of mesotibia’s resting position. **Female** as in male, except anterior pair of teeth more closely placed and located along the anterior margin of the pronotum, and surface of the frons smooth.

##### Etymology.

The Latin adjective *tuberculatus* in its neuter form, reflecting the tubercles on declivital interstriae which are more prominent than in related species.

##### Distribution and biology.

Only known from the holotype locality in Madagascar where it was collected from very thick bark of a fallen *Ficus* branch (Moraceae), 7 cm in diameter. Two pairs were collected at the early stage of tunnel construction, including a mating niche with a short egg tunnel.

##### Comments.

Previous records of *C.flavescens* from Madagascar are most likely *C.tuberculatum* as these two species are very similar. This could also be the case for records of *C.montanum* (recorded as *C.longipilum*), in which the female mainly differs by the more closely set anterior teeth on the pronotum, and the stouter body. These two species are therefore removed from the list of Malagasy species.

#### ﻿ Ctonoxylon montanum

Taxon classificationAnimaliaColeopteraCurculionidae

Eggers

DA32681E-5432-53FE-9D97-3E833B7BF082

Figs 31 , 34 , 37 [female]; 32, 35, 38 [male]

Ctonoxylon montanum Eggers, 1922: 170.
Ctonoxylon longipilum Eggers, 1935: 308, syn. nov.
Ctonoxylon nodosum Eggers, 1940: 236, syn. nov.

##### Links.

https://www.barkbeetles.info/photos_target_species.php?lookUp=7979. https://www.barkbeetles.info/photos_target_species.php?lookUp=2193.

##### Type material.

***Holotype*** female: Kamerun, Buea, XII.10, Hintz, leg. Type 60341 [USNM]. ***Holotype*** of *Ctonoxylonlongipilum*, female: [Tanzania] Mulange, Br. O. Afr. Type 60340 [USNM]. ***Holotype*** of *C.nodosum*, male: [Democratic Republic of the Cogo] Congostaat, Mongbwalu [1.93, 30.05], [1200 m alt.] Mm Scholtz [RMCA].

##### Diagnosis.

Length 3.2–3.6 mm. 2.0–2.1× as long as broad; colour brown. Upper and lower eye parts separated by 1.5× the width of upper part; pronotal eye scraper acutely pointed; a sharp carina running from scraper to procoxa, without associated groove or propleural pit; scutellar shield at level with elytra; striae distinctly impressed, interstriae rounded, interstrial setae curved and bristle-like, variable in length and scattered irregularly within rows, denser on declivity, without ground vestiture; elytral apex in females slightly extended, entire, in males apical interstriae 1 and 2 fused and strongly inflated; resting position of mesotibiae marked on metaventrite.

##### Distribution.

Ivory Coast, Ghana, Nigeria, Cameroon, Democratic Republic of the Congo, Uganda, Kenya, Tanzania

##### New records.

Ghana, Ashanti region, Kwadaso, 320 m., N6.42’ – W1.39’, Dr. S. Endrödy-Younga, mixed light, 25.II.1969 [1, NHMW]; Ghana, Western Region, Ankasa, Nkwanta Camp, 8.6.2005, KB Miller, leg. [1, ZMUB]; Nigeria, Ibadan [7.40, 3.85], 01.11.1964, M.L. Jerath leg. [2, USNM]; Nigeria, Ife, W. State [7.48, 4.48], 01.08.1971, T. Medler [3, USNM]; Cameroon, Libamba, 10 km E of Makak [3.54, 11.09], 11.02.1974, black light, J.A. Gruwell leg. [1, USNM].

##### Comments.

Type specimens of *C.longipilum* and *C.montanum* are near identical and synonymised. The first species has only slightly longer curved elytral setae, but this feature varies between specimens. All specimens of the two nominal taxa are females as characterised by the pair of raised teeth along the anterior margin of the pronotum. Previously Schedl (1972) synonymised *C.nodosum* with *C.longipilum* due to the presumed sexual dimorphism expressed in the inflated elytral apex and the lack of raised teeth along the pronotal margin. Schedl’s view is supported and the synonym *C.nodosum* is therefore moved to *C.montanum*.

**Figures 30–38.**
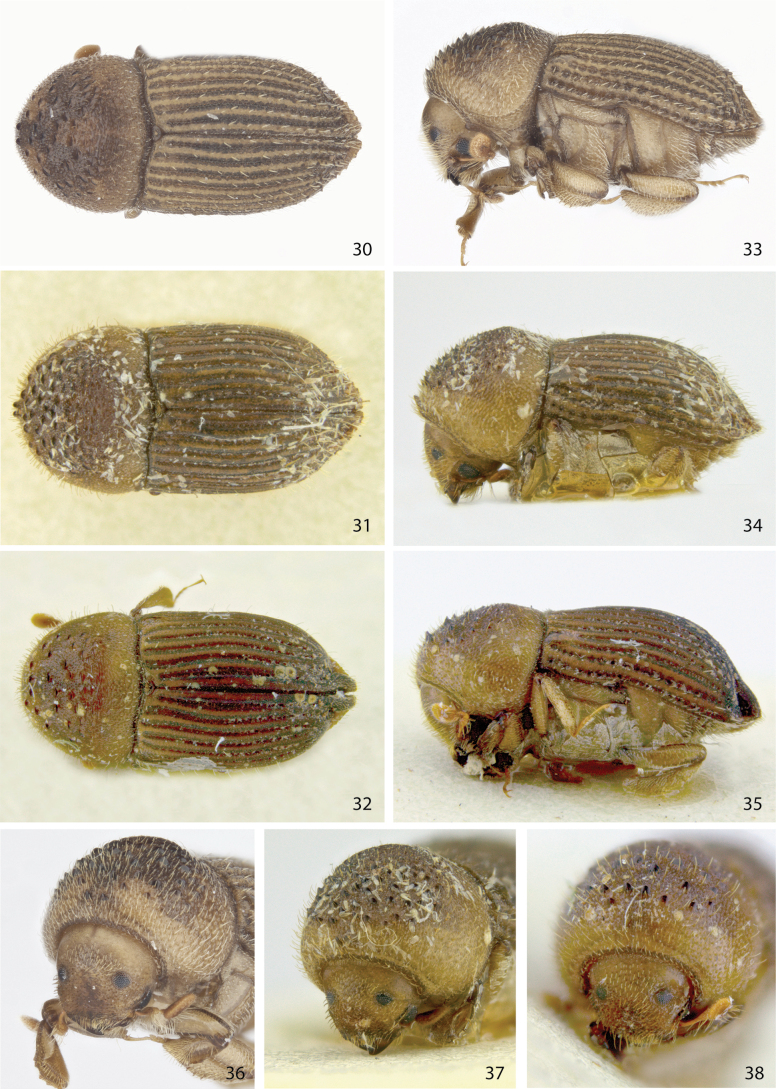
Dorsal, lateral, and front views of **30, 33, 36***Ctonoxylontuberculatum* sp. nov. male holotype **31, 34, 37***Ctonoxylonlongipilum* female (= *C.montanum*), and **32, 35, 38***Ctonoxylonnodosum* male holotype (= *C.montanum*).

The record from Madagascar as *C.longipilum* (see Schedl 1977) is likely a misidentified female specimen of *C.tuberculatum* sp. nov. and is removed from the distribution list. Basically nothing is known about its biology except coming to light at lower to middle altitudes.

#### ﻿ Ctonoxylon camerunum

Taxon classificationAnimaliaColeopteraCurculionidae

Hagedorn

D82C8E5A-3614-5498-956B-18339AA8117E

Figs 39 , 42 , 45

Ctonoxylon camerunum Hagedorn, 1910: 4.
Ctonoxylon fuscum Hagedorn, 1910: 5. Synonym by Eggers 1920.
Ctonoxylon conradti Schedl, 1939: 171, syn. nov.

##### Type material.

***Holotype***: Kamerun [ZMHB]. ***Holotype****C.conradti*, female: [Tanzania] Insel Ukerewi [NHMW].

##### Diagnosis.

Length 3.4–3.8 mm. 2.0–2.1× as long as broad; colour brown. Upper and lower eye parts separated by 1.5× the width of upper part; pronotal eye scraper acutely pointed; a sharp carina running from scraper to procoxa, without associated groove or propleural pit; scutellar shield at level with elytra; striae weakly impressed; interstrial setae sort, bristle-like, dense, confused with similar type of ground vestiture; elytral apex slightly emarginated; elytral suture buckled on disk; resting position of mesotibiae marked on metaventrite.

##### Distribution.

Liberia (new country record), Ivory Coast, Nigeria, Cameroon, Equatorial Guinea, Gabon, Democratic Republic of the Congo, Angola, Tanzania.

##### New records.

Liberia, Suakoko [6.98, -9.54], 28.1–5.5.1952, light trap, Blickenstaff leg (47); Cape Mount [6.72, -11.34], 1940, M. Mann (1) [USNM]; Nigeria, Ife, W. State [7.48, 4.48], 25.03.1969, J.T. Medler [1, USNM]; Cameroon, Yangamo, 100 km NE Bertoua [GIS: 5.00, 14.025], 25.03.1984, Kunze & Nagel, leg. [ZFMK]; 10 km S of Tongo [4.91, 10.77], 02.03.1972, black light, J.A. Gruwell [USNM].

##### Comments.

The holotype of *C.conradti* was compared to a specimen of *C.camerunum* determined by Hagedorn in ZMHB, which is possibly the holotype. The type of *C.conradti* has slightly shorter elytral setae and is less shiny than *camerunum*, but this is very likely within species variation.

#### ﻿ Ctonoxylon cornutum

Taxon classificationAnimaliaColeopteraCurculionidae

Eggers

0402E256-C380-55A7-B485-4A604B7143F2

Figs 40 , 43 , 46

Ctonoxylon cornutum Eggers, 1943: 246.

##### Type material.

***Holotype***: Kamerun, coll Strohmeyer [SDEI].

##### Diagnosis.

Length 3.8–4.1 mm. 1.9–2.0× as long as broad; colour dark brown. Upper and lower eye parts separated by 1.5× the width of upper part; pronotal eye scraper acutely pointed; a sharp carina running from scraper to procoxa, without associated groove or propleural pit; anterior pair of teeth on pronotum much larger than other asperities; scutellar shield at level with elytra; striae weakly impressed; interstrial setae short and bristle-like, dense, confused with similar type of ground vestiture; elytral apex nearly entire, very slightly emarginated; elytral suture buckled a little behind scutellar shield; resting position of mesotibiae marked on metaventrite.

**Figures 39–47.**
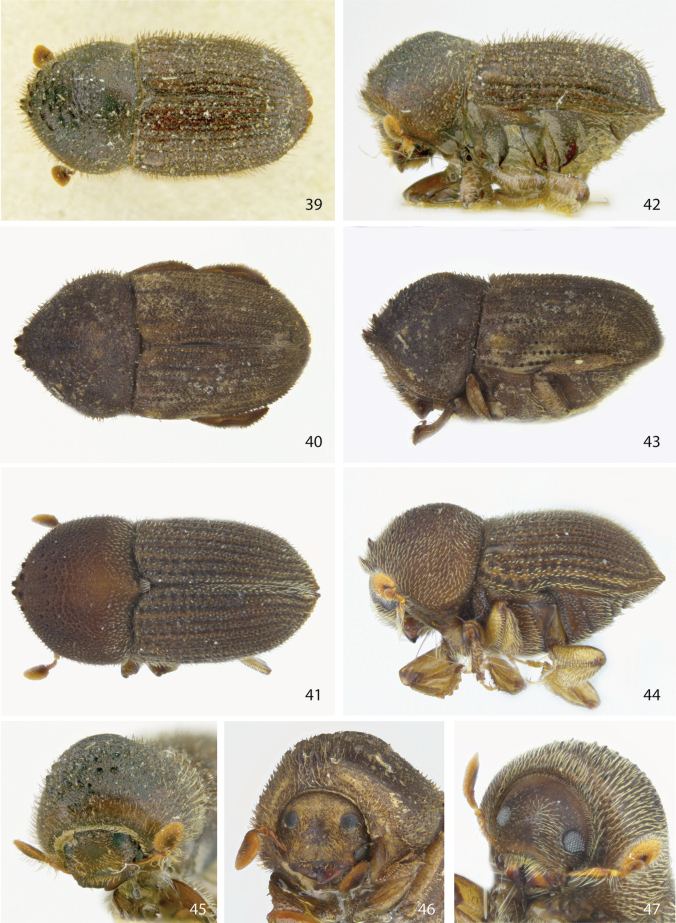
Dorsal, lateral, and front views of **39, 42, 45***Ctonoxyloncamerunum* female **40, 43, 46***Ctonoxyloncornutum* female holotype, and **41, 44, 47***Ctonoxylontorquatum* sp. nov. female holotype.

##### Distribution.

Cameroon.

#### ﻿ Ctonoxylon torquatum sp. nov.

Taxon classificationAnimaliaColeopteraCurculionidae

D629F955-C07E-5E4F-B04A-AEFAA01F95AF

https://zoobank.org/F984EE78-7737-4824-8E00-76B31109A4B1

Figs 41 , 44 , 47

##### Type material.

***Holotype***, Madagascar, Toliara, Sept Lacs [GIS: 23.527, 44.155], MGF076, 02.03.2002, B. Fischer, leg. [CAS]. ***Paratypes***, same data as holotype [CAS (2), NHMW (2), MSUC (2), ZMUB (2)].

##### Diagnosis.

Protibiae with shallow anterior groove of depth < 1/3 width of the tibia; anterior margin of prothorax from coxa to top with an indented row of deep circular pits; scutellar shield and elytral suture with soft white setae; elytral apex with pair of tubercles.

##### Description.

Body length 1.9–2.5 mm, 2.0–2.1× as long as broad; mature colour dark brown. ***Frons*** flattened from just below upper level of eyes to epistoma, surface finely punctured and granulated, central third smooth and impunctate; vestiture of fine short setae, nearly glabrous in middle. Eyes divided, each part separated by 2/3 the size of upper half. Antennal funiculus 7-segmented; club with two asymmetrically and strongly procurved sutures, suture one partly grooved, club segments 1 and 2 each with a dark septum clearly indicated. ***Pronotum*** asperate on central half of anterior three quarters, two front teeth separated by little less than width of a tooth, located at the margin, one additional pair of larger teeth a little behind the front teeth. Lateral anterior margin of prothorax with indented row of deep circular pits running from pronotal teeth to coxa; propleural pit longitudinally elongated, located between coxa and lateral costa on pronotum. ***Scutellar shield*** slightly detached from elytra, slightly sunken, broader than long, with fine white setae. ***Elytral*** striae impressed, punctures round or subquadrate, spaced by less than their diameter; interstriae raised, profile rounded, cuticle rough, vestiture consisting of confused, dense, bristle-like setae, with densely placed soft white setae along a straight elytral suture; apex with a pair of small tubercles. ***Metanepisternum*** with short plumose setae; metaventrite with scattered simple setae. ***Protibiae*** on anterior face with shallow groove for reception of tarsus, ~ 1/3 as deep as width of tibia.

##### Etymology.

The Latin adjective *torquatus* in its neuter form, meaning adorned with a collar, referring to the row of deep pits along the front margin of the prothorax.

##### Distribution and biology.

Only known from a long series of specimens collected at the type locality in the dry forest of south-western Madagascar.

#### ﻿ Ctonoxylon auratum

Taxon classificationAnimaliaColeopteraCurculionidae

Hagedorn

7BD6C725-6FC6-5B3B-A8D6-886250C7908A

Figs 48 , 51 , 54

Ctonoxylon auratum Hagedorn, 1910: 4.

##### Type material.

***Holotype***: Kamerun, Conradt [leg.], coll Kraatz, Hagedorn det. [SDEI].

##### Diagnosis.

Length 2.1–2.2 mm. 2.4–2.5× as long as broad; colour brown. Eyes divided, separated by 2/3 the width of upper part; setae in frons mainly scale-like; anterior margin of pronotum with two teeth fused; anterior margin of prothorax from top to coxa with row of deep circular pits; propleural pit present; elytral vestiture of irregular interstrial rows of scalelike setae mixed with smaller and softer bristle-like setae, appearing somewhat fluffy; elytral apex emarginated; protibiae on anterior face with shallow groove, depth ~ 1/3 of tibia width.

**Figures 48–56.**
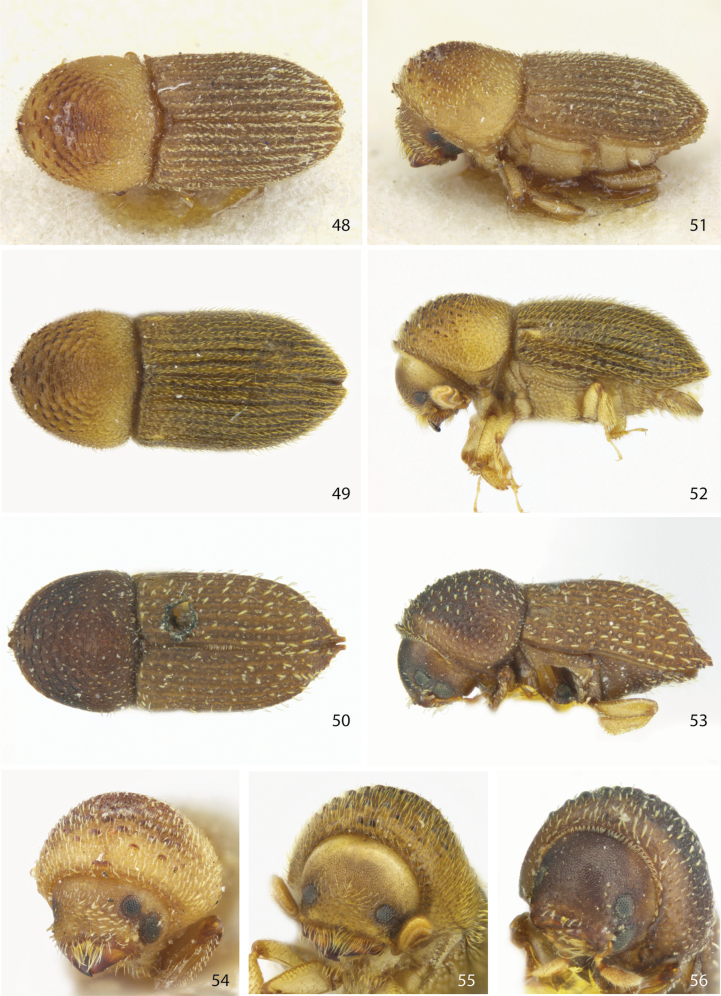
Dorsal, lateral, and front views of **48, 51, 54***Ctonoxylonauratum* female holotype **49, 52, 55***Ctonoxylonpilosum* sp. nov. female holotype, and **50, 53, 56***Ctonoxylonpygmaeum* female syntype [ZMHB].

##### Distribution.

Cameroon, Democratic Republic of the Congo.

##### Remarks.

The female holotype of this species is located in Muncheberg [SDEI], not Berlin as stated in Wood and Bright (1992).

#### ﻿ Ctonoxylon pilosum sp. nov.

Taxon classificationAnimaliaColeopteraCurculionidae

1A1ACC8E-0E89-5A86-A104-2A70A2B48115

https://zoobank.org/4F557860-8B61-4BC3-8B06-921E1D3916C8

Figs 49 , 52 , 55

##### Type material.

***Holotype***, female: Cameroon, Ekande near Limbe [GIS: 4.081, 9.172], 1100 m alt., 18.11.2007, ex thin liana, A. Breistøl, leg. [ZMUB]. ***Allotype*** [ZMUB] and one ***paratype*** [NHMW]: same data as holotype.

##### Diagnosis.

Protibiae with shallow anterior groove of depth < 1/3 the width of the tibia; anterior margin of prothorax from coxa to top with indented row of deep circular pits; elytral interstriae with dense, soft, golden ground vestiture in addition to longer, curved, bristle-like main setae.

##### Description.

Body length 1.4–1.8 mm, 2.2–2.4× as long as broad; colour yellowish brown. ***Frons*** flattened from just below upper level of eyes to epistoma, surface finely punctured below, reticulate above, vestiture consisting of sparse, fine, short setae. Eyes divided, each part separated by 2/3 the size of upper half. Antennal funiculus 7-segmented; club with two asymmetrically and strongly procurved sutures, suture one partly grooved with a dark partial septum along the front margin of the suture. ***Pronotum*** coarsely asperate on central two-thirds of anterior two-thirds, two front teeth at margin subcontiguous, not particularly larger than other asperities. Anterior lateral margin of prothorax with indented row of deep circular pits running from the anterior pronotal teeth to coxa; a round propleural pit located between coxa and lateral costa on pronotum. ***Scutellar shield*** flush with elytra, with fine golden dorsal setae. ***Elytral*** striae impressed, punctures round, irregularly sized and spaced; interstriae flat to slightly rounded, vestiture consisting of irregular rows of curved bristle-like setae, with ground vestiture consisting of more densely placed, soft, golden, hair-like setae; elytral suture straight, apex weakly emarginated. ***Metanepisternum*** and upper metaventrite with short plumose setae, simple and longer setae elsewhere. ***Protibiae*** on anterior face with shallow groove for reception of tarsus, ~ 1/3 as deep as width of tibia.

**Male** nearly identical to female, except frons more distinctly impressed below upper level of eyes, and the anterior pair of pronotal teeth is located just slightly behind the front margin.

##### Etymology.

The Latin adjective *pilosus* in its neuter form, meaning hairy, referring to the dense ground vestiture of fine, short, golden setae.

##### Distribution and biology.

Only known from the type locality in Cameroon where specimens were collected from a climbing plant, ~ 2 cm in diameter.

#### ﻿ Ctonoxylon pygmaeum

Taxon classificationAnimaliaColeopteraCurculionidae

Eggers

4A630D9C-F61C-5B12-80B1-AC0DAE0E0EE7

Figs 50 , 53 , 56

Ctonoxylon pygmaeum Eggers, 1920: 39.

##### Type material.

***Syntypes***, females: Kamerun, Soppo, 800 m., XII 1912, v. Rothkirch S.G. [ZMHB, NHMW].

##### Diagnosis.

Length 1.6–1.7 mm. 2.2–2.4× as long as broad; colour dark brown. Eyes divided, separated by half the width of upper part; frons nearly glabrous, reticulate; pronotal asperities broadly distributed on anterior three-quarters; lateral anterior margin of prothorax from top to coxa with row of shallow irregular pits; propleural pit present just above coxa; elytral vestiture of regular interstrial rows of scalelike setae only; elytral apex expanded by pair of elongated prong-like tubercles; protibiae on anterior face with deep groove.

##### Distribution.

Cameroon.

#### ﻿ Ctonoxylon caudatum

Taxon classificationAnimaliaColeopteraCurculionidae

Schedl

0A221312-3A8E-555A-954C-1ED92C7BFD35

Figs 57 , 60 , 63

Ctonoxylon caudatum Schedl, 1971: 8.

##### Type material.

***Holotype***, male: [Democratic Republic of the] Congo Belge, Stanleyville [Kisangani], 19.6.1952, K.E. Schedl [NHMW].

##### Diagnosis.

Length 3.5 mm. 2.2× as long as broad; colour dark brown and black. Eyes divided, separated by the size of upper part; head black, frons reticulate, with scattered short setae; pronotal asperities broadly distributed on anterior three-quarters; lateral anterior margin of prothorax from middle part to coxa with shallow irregular groove; propleural pit presumably present; elytral vestiture of multiple confused rows of short interstrial scale-like setae; elytral apex expanded by pair of elongated prong-like tubercles; protibiae on anterior face with shallow groove of depth 1/3 the width of tibia.

##### Distribution.

Democratic Republic of the Congo.

#### ﻿ Ctonoxylon crenatum

Taxon classificationAnimaliaColeopteraCurculionidae

Hagedorn

769F841C-36A1-541E-9128-2F2A982B2701

Figs 58 , 61 , 64

Ctonoxylon crenatum Hagedorn, 1910: 5.

##### Type material.

***Holotype***, male: Kamerun, Conradt [leg.], coll Kraatz, Hagedorn det. [SDEI].

##### Diagnosis.

Length 2.4–2.5 mm. 2.1× as long as broad; colour brown. Eyes divided, separated by slightly more than the size of upper part; frons lightly punctured, reticulate, glabrous; pronotum strongly domed, summit near posterior margin; pronotal asperities low, broad, subcontiguous, distributed on anterior three-quarters; lateral anterior margin of prothorax from middle part to coxa with shallow irregular groove; propleural pit presumably present; elytra glabrous, shiny, striae impressed; elytral apex expanded by pair of elongated prong-like tubercles.

##### Distribution.

Cameroon, Republic of the Congo (new country).

##### New record.

Republic of the Congo, Dimonika, Mayumbe [GIS: -4.46, 12.45] [1, NHMW].

#### ﻿ Ctonoxylon kivuensis

Taxon classificationAnimaliaColeopteraCurculionidae

Schedl

B04D4084-EE18-5A23-948B-68DD347ADA74

Figs 59 , 62 , 65

Ctonoxylon kivuensis Schedl, 1957: 44.

##### Type material.

***Holotype***, male: [Democratic Republic of the] Congo Belge, Kivu, Mulungu, 2.VIII.1952, ex *Popowia*, KE Schedl, leg. [RMCA, paratype in NHMW].

**Figures 57–65.**
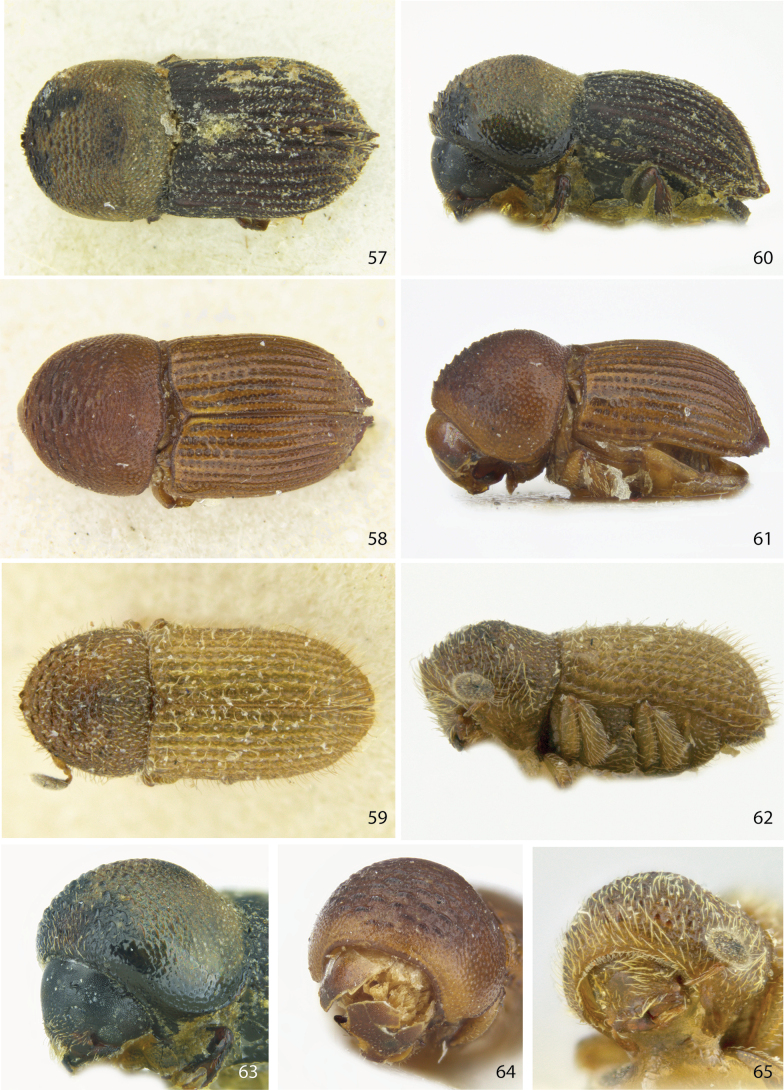
Dorsal, lateral, and front views of **57, 60, 63***Ctonoxyloncaudatum* male holotype **58, 61, 64***Ctonoxyloncrenatum* male holotype, and **59, 62, 65***Ctonoxylonkivuensis* male holotype.

##### Diagnosis

**(male).** Length 1.6 mm. 2.2× as long as broad; colour brown. Eyes divided, separated by slightly more than the size of upper part; antennal club setose, sutures obscure; male frons slightly impressed, smooth and glabrous in centre, with fine soft setae closer to eyes and vertex; pronotum roughly punctured, with sharp asperities on anterior half, anterior pair of teeth small, located behind front margin; lateral anterior margin of prothorax from middle part to coxa with shallow irregular groove; propleural pit round, deep; elytral striae impressed, punctures large, separated by their diameter or less; vestiture consisting of irregular rows of long, soft bristle-like setae; elytral suture slightly buckled at midlength, apex entire.

##### Distribution.

Democratic Republic of the Congo.

##### Biology.

Only known from the medium altitude (732 m) type locality in the Congo basin, breeding in a *Popowia* branch (Annonaceae).

#### ﻿ Ctonoxylon quadrispinum sp. nov.

Taxon classificationAnimaliaColeopteraCurculionidae

84D97D21-5B46-579A-B80C-5D193A9A5DC9

https://zoobank.org/7345B8EE-8158-414F-860F-3A7F5DE6B9CD

Figs 66 , 69 , 72

##### Type material.

***Holotype***, female: Madagascar, Ankarafantsika NP [GIS: -16.264, 46.828], 200 m alt., 8.5.2015, ex liana, B. Jordal, leg. [ZMUB]. ***Paratypes***: same data as holotype [NHMW, ZMUB].

##### Diagnosis.

Anterior teeth on the pronotum quadrifid; lateral anterior margin of prothorax with elongated groove; propleural pit present; scutellar shield detached; elytral suture strongly curved just behind scutellar shield, a little buckled further behind.

##### Description.

**Female.** Body length 2.6–3.0 mm, 2.1× as long as broad; immature colour pale brown. ***Frons*** convex, surface reticulate, vestiture consisting of sparse, short setae. Eyes divided, each part separated by 2/3 the size of upper half. Antennal funiculus 7-segmented; club with two asymmetrically and strongly procurved sutures, suture one more distinctly marked, with a dark partial septum along its front margin. ***Pronotum*** broadly asperate on anterior two-thirds, anterior margin with pair of two bifid raised teeth (quadrifid). Anterior lateral margin of prothorax with pointed eye scraper at level of eyes, a deep groove from this point to coxa; a transverse propleural groove located between coxa and lateral costa on pronotum. ***Scutellar shield*** detached from elytra. ***Elytral*** suture strongly curved immediately behind scutellar shield and slightly buckled at midlength; striae strongly impressed, punctures round, large and deep, spaced by their diameter or less; interstriae raised, vestiture consisting of irregular and partly confused rows of short scale-like setae, with ground vestiture consisting of shorter setae of the same type and density; apex slightly emarginated. ***Metanepisternum*** and metaventrite with scant simple setae; last abdominal ventrite with small concavity. ***Protibiae*** on anterior face with groove for reception of tarsus as deep as width of tibia.

**Male** nearly identical to female, except frons impunctate and glabrous in a narrow band from epistoma to upper level of eyes, front teeth along the pronotal margin very slightly behind the position in females, and last abdominal ventrite flat.

**Figures 66–74.**
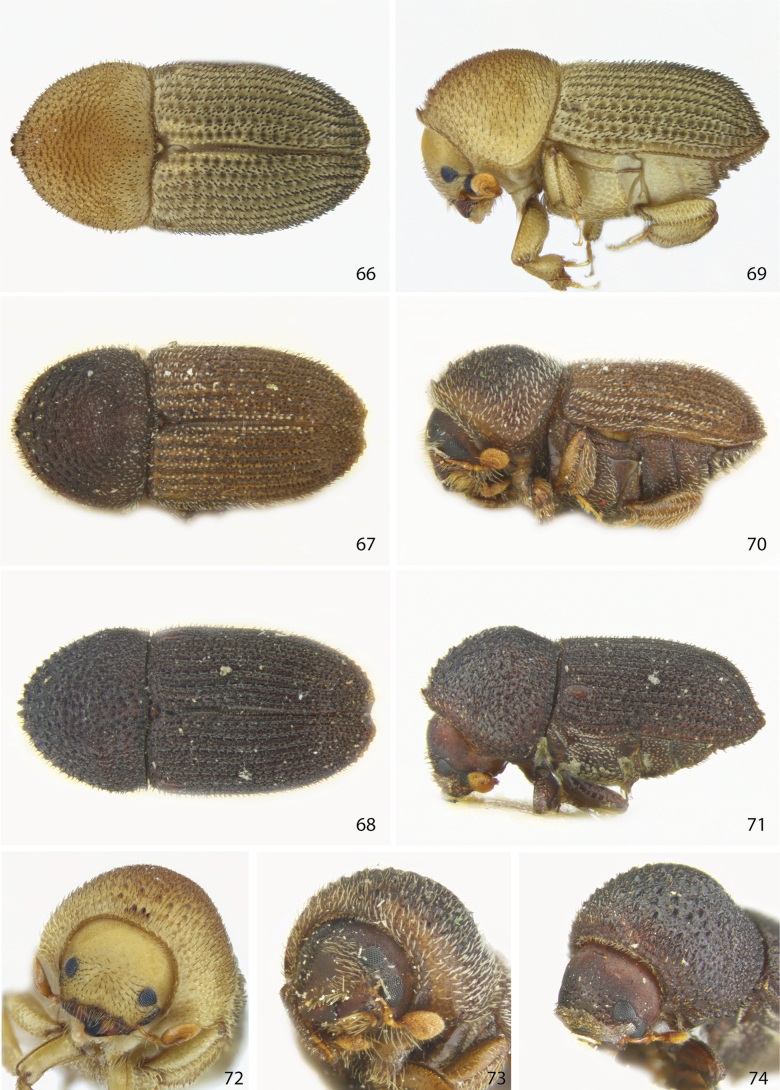
Dorsal, lateral, and front views of **66, 69, 72***Ctonoxylonquadrispinum* female holotype **67, 70, 73***Ctonoxylonmethneri* male, and **68, 71, 74***Ctonoxylonatrum* female holotype.

##### Etymology.

Composed by the Latin prefix *quadri*- meaning four, and the Latin adjective *spinum* in its neuter form derived from *spina*, meaning thorn, referring to the four sharp teeth along the front margin of the pronotum.

##### Distribution and biology.

Only known from the type locality in Madagascar where broods containing pupae and tenerals, but not parents, were collected from a liana 4 cm in diameter, with thick bark. Egg tunnels were biramous and cut transversely to the grain. Brood sizes ranged from 50–60 (*n* = 20).

#### ﻿ Ctonoxylon methneri

Taxon classificationAnimaliaColeopteraCurculionidae

Eggers

14DDD684-CC1B-5E5E-A5A7-0BA5EFD6D23D

Figs 67 , 70 , 73

Ctonoxylon methneri Eggers, 1922: 170.
Ctonoxylon griseum Schedl, 1941: 389, syn. nov.
Ctonoxylon hamatum Schedl, 1941: 400, syn. nov.

##### Type material.

***Holotype*** (‘type’): [Tanzania] Udzungwa-Berge1300–1600 m., 26.XI.1912, Methner, coll. [ZMHB, not found]. ***Holotype*** of *C.hamatum* male: [Kenya] Nairobi [NHMW]. ***Holotype*** of *C.griseum*, female: [Kenya] Brit. O. Africa, Kikuyu [-1.27, 36.68], E. Thomas [NHMW].

##### Diagnosis.

Length 2.3–4.2 mm. 2.1–2.3× as long as broad; colour dark brown, dorsal setae variegated. Male frons flat, smooth, impunctate and glabrous within a triangular pattern of short setae near eyes and epistoma; female frons finely granulated with short setae; eye parts separated by almost the size of upper half; anterior lateral margin of prothorax with elongated groove near front of coxa; propleural pit present just above coxa; scutellar shield detached from elytra; elytral suture slightly buckled at midlength; apex emarginated; interstrial vestiture consisting of confused rows of variegated scale-like setae of similar length and colour as the ground vestiture.

##### Distribution.

Kenya, Tanzania, South Africa.

##### New records.

South Africa, E. Cape Prov., Katberg [GIS: -32.479, 26.673], 01.11.1933, R.E. Turner, leg. [2, NHMUK]; W. Cape province, Knysna, Diepwalle [GIS: -33.957, 23.152], 7.11.2006, ex *Oleacapensis* trunk, B. Jordal, leg; same data except Goudveld, Krisjan Se-Nek [GIS: -33.913, 22.948], 5.11.2006; Goudveld, Woodcutters [GIS: -33.927, 22.976], 4.11.2006; Gouna, Grootdrai [GIS: -33.946, 23.054], 6.11.2006; Nature’s Valley [GIS: -33.965, 23.562], 8.11.2006; Tsitsikamma, Goesa walk [GIS: -33.983, 23.887], 12.11.2006; Tsitsikamma, Platboos walk [GIS: -33.980, 23.910], 14.11.2006 [all in ZMUB]; Kenya, Ngong Forestry [-1.31, 36.73], 12.01.1968, Malaise trap, Krombein & Spangler leg. [1, USNM].

##### Biology.

This species is exclusively associated with black ironwood, *Oleacapensis* (Oleaceae) where it is usually present whenever branches and trees are down. Recent field studies in the Knysna forests in the Cape region indicated no particular preference for breeding material size, ranging from 2-cm thick branches to the largest tree trunks of > 60 cm diameter. It breeds in the same host tree as *Lanurgusjubatus* Jordal, 2021, and *Lanurgusxylographus* Schedl, 1961, but even though it is found in the same forest localities, *C.methneri* only co-occurred with these species in the same tree one out of ten collecting events. Brood size was not smaller in the thinnest branches examined and broods with larvae or older stages ranged from 17–48 (Table 5). Very few broods with larvae had a male parent present with the female, and then only at very early larval stage. In the large majority of dissected broods, the male left before all eggs were laid or hatched. The female left her offspring before pupal stage. Egg tunnels were always cut transversely to the wood grain and eggs deposited vertically in deep pits packed with frass.

##### Remarks.

Some specimens of *C.methneri* differ from those of *C.hamatum* by having slightly shorter setae on interstriae 1 and 2 but this appears to be intraspecific variation. The differences in frons and pronotum between the types of *C.hamatum* and *C.methneri* on one side, and *C.griseum*, is due to sexual dimorphism otherwise typical for the genus.

#### ﻿ Ctonoxylon atrum

Taxon classificationAnimaliaColeopteraCurculionidae

Browne stat. rev.

F041320C-A74B-5813-9E3B-00876F657130

Figs 68 , 71 , 74

Ctonoxylon atrum Browne, 1965: 190.

##### Type material.

***Holotype***, male: Nigeria, Idanre [GIS: 7.12, 5.10], 30.9.1964, ex *Canthium* [NHMUK].

##### Diagnosis.

Length 3.4 mm. 2.1–2.2× as long as broad; colour pitch black. Male and female frons slightly impressed and impunctate on lower half, finely granulated above with short scattered setae; eye parts separated by 2/3 the size of upper half; anterior lateral margin of prothorax with elongated groove near procoxa; propleural pit present just above procoxa; pronotum with large and deep irregular punctures; elytral suture buckled at midlength, strial punctures longitudinally elongated, subquadrate; interstriae rough, with irregular punctures and rugosities; elytral apex emarginated; interstrial vestiture of confused rows of short, black, scale-like setae.

##### Distribution.

Ghana, Cameroon (new country).

##### New record.

Cameroon, Limbe, Ekande [GIS: 4.081, 9.172], 19. Nov. 2007, ex water liana, B. Jordal, leg [1, ZMUB].

##### Remarks.

This species is rather similar to *C.methneri* and was synonymised with *C.hamatum* by Schedl (1970). However, *C.atrum* is darker, with shorter elytral setae, the strial punctures are narrowly elongated and more separated, and the pronotal punctures are larger. It is genetically clearly separated from *C.methneri* and instead forms a sister relationship with *C.quadrispinum* sp. nov. (Fig. 11).

#### ﻿ Ctonoxylon acuminatum

Taxon classificationAnimaliaColeopteraCurculionidae

Schedl

F9DDD8EC-7DFC-5E12-B59C-6E8D82210980

Figs 75 , 77 , 79

Ctonoxylon acuminatum Schedl, 1957: 45.

##### Type material.

***Holotype***, male: [Democratic Republic of the] Congo Belge, Yangambi, 9.IX.1952, ex *Clitandrastaudtii*, KE Schedl, leg. [RMCA, paratype in NHMW].

##### Diagnosis.

Length 2.6 mm. 2.3× as long as broad; colour pale brown. Male frons reticulate with short fine setae; eye parts separated by the size of upper half; anterior lateral margin of prothorax with obtuse eye scraper, from this point to coxa with elongated groove; propleural pit present; elytral suture slightly buckled at midlength; apex entire, slightly extended and upcurved in lateral view; interstrial vestiture of regular rows of long bristle-like setae.

**Figures 75–80.**
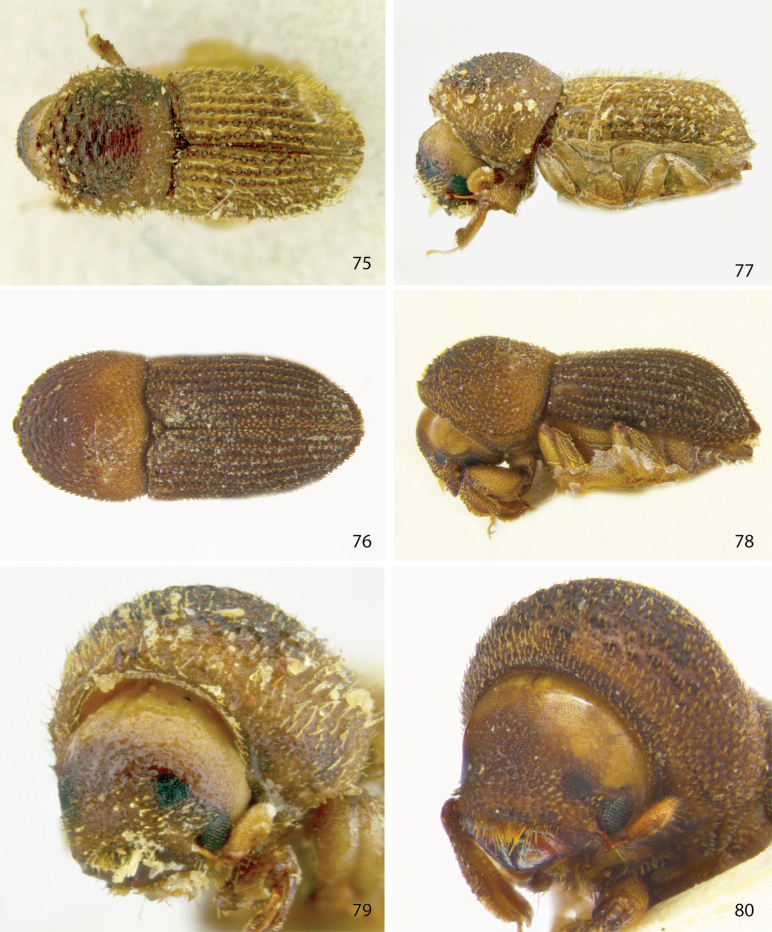
Dorsal, lateral, and front views of **75, 77, 79***Ctonoxylonacuminatum* female holotype, and **76, 78, 80***Ctonoxylonspathifer* male.

##### Biology.

One host plant is known, *Orthopichoniavisciflua* (K.Schum. ex Hallier f.) Vonk (Apocynaceae), a thin climbing plant (Schedl 1961).

##### Comments.

Superficially similar to *C.kivuensis* but has shorter setae and the elytral apex in lateral view has a slightly curved extension (Fig. 77).

#### ﻿ Ctonoxylon spathifer

Taxon classificationAnimaliaColeopteraCurculionidae

Schedl

EAC73FE2-757A-51D9-A307-FD0CE7943729

Figs 76 , 78 , 80

Ctonoxylon spathifer Schedl, 1951: 39.

##### Type material.

***Syntypes***: [Ivory Coast] Cote d’Ivoire, Reserve du Banco [5.39, -4.05] [MNHN, NHMW].

##### Diagnosis.

Length 2.6–3.1 mm. 2.3–2.4× as long as broad; colour reddish brown. Eyes divided, separated by the size of upper part; antennal club setose, sutures obscure; male frons roughly punctured, glabrous in middle, with short coarse setae close to the eyes; pronotum with dense subconfluent asperities on anterior two-thirds, a fused pair of teeth just behind front margin; lateral anterior margin of prothorax from middle part to coxa with shallow irregular groove; propleural pit round, deep; elytral striae impressed, punctures large, deep, narrowly separated; vestiture on interstriae consisting of densely confused, short, scale-like setae; elytral suture straight, apex narrowly rounded, entire.

##### Distribution.

Ivory Coast, Ghana (new country), Tanzania.

##### New record.

Ghana, Samreboi [5.61, -2.55], ex *Trichiliarubescens*, 10.IX.1962, F.G. Browne, leg. [2, NHMUK].

##### Biology.

Known to breed in *Oleawelwitschii* (Oleaceae), *Pachylobusdeliciosus* (Burseraceae), *Strombosiapostulata* (Olacaceae), and *Trichiliarubescens* (Meliaceae), an unusually broad assemblage of host plants for a true bark beetle.

#### ﻿ Ctonoxylon amanicum

Taxon classificationAnimaliaColeopteraCurculionidae

Hagedorn

BCCB6796-B046-52AC-AD30-044C92D32B07

Figs 81 , 84 , 87

Ctonoxylon amanicum Hagedorn, 1912: 42.
Ctonoxylon intermedium Schedl, 1971: 10, syn. nov.

##### Type material.

***Holotype***: [Tanzania] D.O. Afrika, Amani [ZMHB]. ***Holotype***, *C.intermedium*: Kamerun, C. Conradt leg. [NHMW].

##### Diagnosis.

Length 1.6–1.9 mm. 2.2–2.4× as long as broad; colour brown. Female frons slightly impressed on central lower half, glabrous and impunctate in middle, with short stiff setae around central area; eye parts narrowly separated by half the size of upper eye part; lateral anterior margin of prothorax with obtuse eye scraper, further below near coxa with short elongated groove; propleural pit present above coxa; elytral suture a little buckled at midlength; apex entire; interstrial vestiture consisting of regular rows of narrowly spatulate setae; posterior part of the metaventrite with unusually long, broad setae.

##### Distribution.

Cameroon, Tanzania.

New record: Tanzania, Morogoro prov. Udzungwa [GIS: -7.85, 36.89], ex liana, 29.6.2010, B. Jordal leg. [ZMUB]. Cameroon, Limbe, Ekande [GIS: 4.081, 9.172], 1100 m alt., ex thin liana, 18.11.2007, A. Breistøl, leg.

##### Biology.

One specimen was collected in each of two localities, in countries with type localities for the two synonymised species *C.amanicum* and *C.intermedium*. Both specimens were tunnelling in thin lianas, one under bark and one in pith of a stem nodule in which only a short irregular tunnel was made.

**Figures 81–89.**
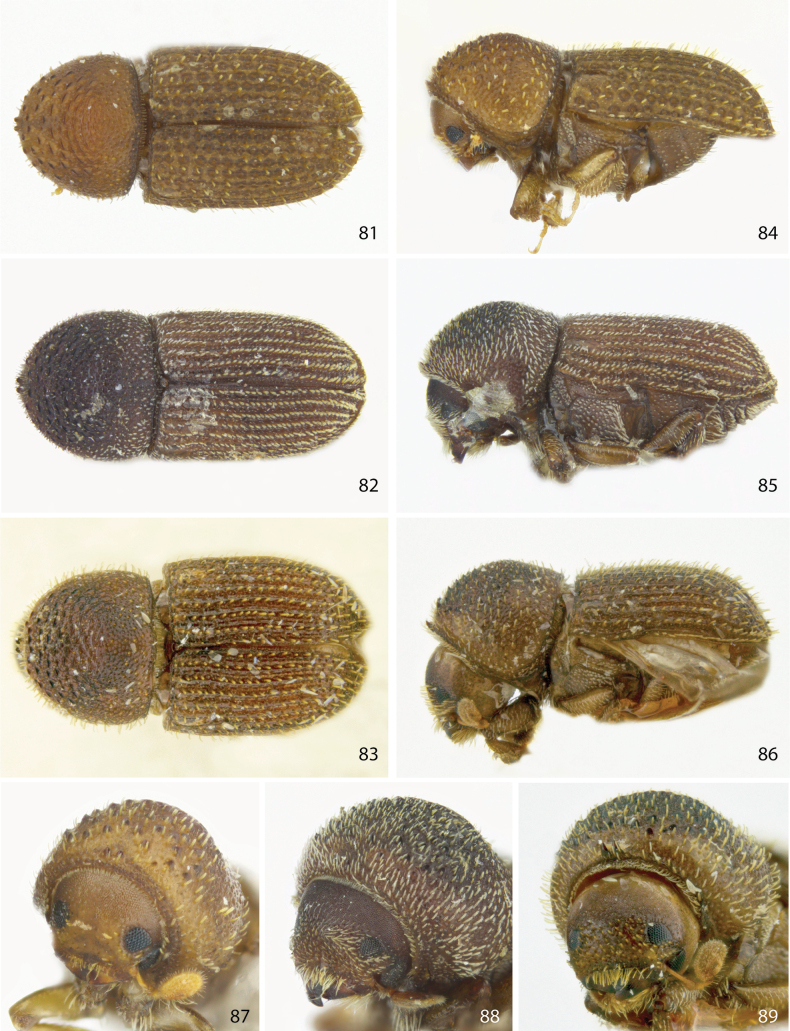
Dorsal, lateral, and front views of **81, 84, 87***Ctonoxylonamanicum* female **82, 85, 88***Ctonoxylonuniseriatum* female, and **83, 86, 89***Ctonoxylonspinifer* female.

##### Comments.

The holotype of *C.amanicum* was supposed to be lost in the museum of Hamburg but was rediscovered in Berlin (ZMHB). The holotype of *C.intermedium* is identical to *C.amanicum* in all important characteristics and therefore synonymised.

#### ﻿ Ctonoxylon uniseriatum

Taxon classificationAnimaliaColeopteraCurculionidae

Schedl

E88CE4F2-DD99-5E4C-88D0-2957E925B71E

Figs 82 , 85 , 88

Ctonoxylon uniseriatum Schedl, 1965: 114.
Ctonoxylon capensis Schedl, 1971: 8, synonym by Beaver 2011.
Cryphalostenus erratium Schedl, genus et species nomen nudum.

##### Type material.

***Holotype***: [Namibia] Deutsch S.W. Afrika [NHMW]. ***Holotype*** of *C.capensis*: [South Africa] Umgeberge, Cape Town, 1899 [NHMW].

##### Diagnosis.

Length 2.3–2.8 mm. 2.3–2.4× as long as broad; colour dark brown. Male frons slightly inflated, smooth and impunctate with short setae near eyes and epistoma; female frons finely punctured with short erect setae; eye parts separated by the size of upper half; lateral anterior margin of prothorax with short elongated groove near front of procoxa; propleural pit large; scutellar shield detached from elytra; elytral suture a little buckled at midlength; apex entire; punctures on discal interstriae obscure, interstriae 1–7 with uniseriate, spatulate, curved setae, interstriae 8–10 sometimes with variably confused rows of setae, on declivital interstriae 1 and 2 setae partly confused, directed sideways; posterior metaventrite with unusually broad and long setae.

##### Distribution.

Namibia, South Africa.

##### New records.

South Africa, W. Cape province, 10 km N Hoekwil, Woodville [GIS: -33.933, 22.639], 1.Nov.2006; Knysna, Diepwalle [GIS: -33.957, 23.152], 3.11.2006 and 7.11.2006; Knysna, Goudveld, Krisjan Se-Nek [GIS: -33.913, 22.948], 5.11.2006, all collections ex liana, B. Jordal, leg. [ZMUB].

##### Biology.

A common species found exclusively in thin lianas ~ 1 cm in diameter. Females were excavating longitudinal tunnels in the pith and wood of rather fresh material which still exuded latex. Males had left the female either before egg laying or just after the first eggs were laid. Broods were small, with 3–8 eggs per female (Table 5).

##### Remarks.

A specimen in NHMW marked as the holotype of *Cryphalostenuserratium* det. Schedl is not published. It is clearly the same species as *C.uniseriatum*.

#### ﻿ Ctonoxylon spinifer

Taxon classificationAnimaliaColeopteraCurculionidae

Eggers

17ED380A-5802-5082-8897-C93779E72F7B

Figs 83 , 86 , 89

Ctonoxylon spinifer Eggers, 1920: 39.
Ctonoxylon setifer Eggers, 1920: 39, syn. nov.

##### Links.

https://www.barkbeetles.info/photos_target_species.php?lookUp=7983.

##### Type material.

***Lectotype***, male of *C.spinifer*: Kamerun, Soppo, 1912, v. Rothkirch leg. [USNM]; ***paratype***, female, same data [NHMW]. ***Holotype****C.setifer*: [Tanzania] Amani [lost]; ***paratype*** [NHMW].

##### Diagnosis.

Length 2.2–2.8 mm. 2.2–2.3× as long as broad; colour brown. Female frons sparsely punctured, lightly granulated with short erect setae; male frons flat, smooth, impunctate and glabrous on central third, with longer setae near eyes and epistoma; eye parts separated by the size of upper half; anterior lateral margin of prothorax with faint eye scraper, below this point with a short elongated groove near front of procoxa; propleural pit present above coxa; scutellar shield detached from elytra; elytral suture a little buckled at midlength; apex entire; interstrial vestiture of mainly regular rows of bristle-like erect setae, interstrial punctures on both disc and declivity dense, distinct; posterior metaventrite with unusually broad and long setae.

##### Distribution.

Burkino Faso (new country record), Ivory Coast, Cameroon, Democratic Republic of the Congo; Kenya, Tanzania, Madagascar (new country record).

##### New records.

Burkina Faso, Foret de Boulon [10.343, -4.510], 270 m, piege interception (1), piege limeneux (1), 9.7.2006, F. Genier, leg. [CMNC]; Cameroon, Adamoua, 20 km S. Minim [6.49, 12.52], 1200 m alt., 8.3.1982, Flacke & Nagel, leg. [1, ZFMK]; Tanzania, Udzungwa National Park HQ, Mang’ula [GIS: -7.845, 36.880], 200 m alt., ex liana, 9.11.2009 and 29.6.2010, B. Jordal, leg.; Mang’ula [GIS:-7.850, 36.883], ex liana, 14.11.2009, B. Jordal, leg. [ZMUB]; Morogoro, Kimboza Forest Reserve [-7.023, 37.806], S.S. Madoffe, leg. [1, NHMUK]; Madagascar, Reserve speciale de l’Ankarana, 22.9 km SW Anivoran [-12.93, 49.16], B. Fischer, leg. [1, CAS].

##### Biology.

Specimens were dissected from the pith of thin lianas, 0.6–1.0 cm diameter, still exuding latex.

##### Comments.

The co-types (paratypes) of *C.spinifer* are identical to those of *C.setifer*. Because the holotype of *C.setifer* is lost, and using the principle of first reviser, the name *spinifer* is given priority. Eggers’ descriptions are very similar and not useful to distinguish specimens. There is some variation within series of various other collections, particularly in the regularity of interstrial rows of setae but this variation follows no predictable pattern. This species is very similar to *C.uniseriatum* but differs by the erect interstrial setae, particularly on the declivity where setae are not directed sideways as in *uniseriatum*, by the densely placed interstrial punctures, and in males also by the less inflated upper central frons. Genetic data supported a sister relationship to *C.amanicum* instead of *C.uniseriatum* (Fig. 11).

A single specimen is recorded from near the north-west coast of Madagascar. This is not too surprising given the broad Afrotropical distribution of this species.

### ﻿Removed from taxon list

***Ctonoxylonalutaceus* (Schaufuss, 1897), nom. dub.**

The type for this taxon is lost from the Hamburg Museum collection. It was examined by Eggers (1922) who stated that the specimen could not be studied due to the partial inclusion in resin. It is therefore not possible to validate species status and is therefore a nomen dubium.

### ﻿Identification key to species

**Table:** 

1	Near anterior lateral margin of the prothorax with a sharp carina running down to front of procoxa, without pleural pit above the coxa (Fig. 1); anterior part of metaventrite with a flattened field and swollen posterior margin demarcating position of resting mesotibia (Fig. 8); prothoracic eye scraper usually acuminate (Fig. 1) (except for one small species, see next couplet)	**2**
–	Near anterior lateral margin of the prothorax with with a deep elongated groove (Fig. 3) or row of deep circular pits (Fig. 4) running down towards procoxa; a distinct propleural pit or transverse groove present between coxa and pronotum (Fig. 5); metaventrite not modified; prothoracic eye scraper either a round faint nodule or entirely absent	**10**
2	Elytral interstriae with sparse but very long, curved setae, longer than width of metatibia; upper and lower eye parts separated only by scapus thickness; anterior lateral margin of prothorax without eye scraper; body length 1.5 mm	***C.hirtellum***
–	Elytral interstriae with various types of vestiture; upper and lower eye parts separated by more than width of upper eye; eye scraper large and triangular; body size much larger, length 2.2–4.2 mm	**3**
3	Elytral apex in dorsal view entire, pointed in both sexes, in males strongly inflated	***C.montanum***
–	Elytral apex emarginated	**4**
4	All elytral vestiture bristle- or scale-like; setae on posterior part of metaventrite narrow scales	**5**
–	Main interstrial setae fine bristles or hair-like; setae on metaventrite hair-like	**6**
5	Elytral ground vestiture consisting of very short scales; anterior median pair of teeth on pronotum large and strongly raised, each tooth ~ the size of 1/2 an upper eye	***C.cornutum***
–	All vestiture longer than width of interstriae; anterior pronotal teeth of normal size	***C.camerunum***
6	Elytral ground vestiture of the same length and confused with the main row of setae	***C.festivum***
–	Main interstrial setae much longer than ground vestiture or ground vestiture absent (*flavescens* group)	**7**
7	Interstriae 1 and 3 on declivity with sharp tubercles almost as large as one tarsomere; interstriae 2 less raised near apex (Madagascar)	***C.tuberculatum* sp. nov.**
–	Declivital interstriae with much smaller granules; interstriae 2 and 3 equal (Africa)	**8**
8	Apical margin of the elytra nearly smooth or with only few fine granules; elytral ground vestiture consisting of fine short hair-like setae	***C.hirsutum***
–	Apical margin of the elytra granulated; elytral ground vestiture absent	**9**
9	Elytral apex almost entire, with very shallow emargination, in lateral view declivity short and straight	***C.bosqueiae***
–	Elytral apex deeply emarginated, in lateral view apex slightly extended	***C.flavescens***
10	Just behind the anterior lateral margin of prothorax with a row of deep circular pits reaching near front of procoxae	**11**
–	Just behind the anterior lateral margin of prothorax with an elongated groove (sometimes with many small confluent pits squeezed into a groove)	**13**
11	Pronotum with small asperities, the majority of these are smaller than the width of a funicular segment; elytral apex at suture with two small tubercles; declivital interstriae 1 with dense fine pale-coloured setae	***C.torquatum* sp. nov.**
–	Pronotal asperities ~ as broad as the scutellar shield; elytral apex rounded or weakly emarginated; all elytral setae of the same golden colour	**12**
12	Setae in frons and elytral interstriae mainly spatulate; anterior margin of pronotum with a single fused tooth	***C.auratum***
–	Setae in frons and elytral ground vestiture hair-like, main interstrial setae of curved fine bristles; two teeth at anterior margin of pronotum separated	***C.pilosum* sp. nov.**
13	Elytral apex extended into a pair of short contiguous spines (Figs 50, 57, 58)	**14**
–	Elytral apex rounded or emarginated	**16**
14	Elytra mainly glabrous	***C.crenatum***
–	Elytral interstriae with setae	**15**
15	Elytral interstriae with multiple confused rows of short setae, each seta shorter than width of interstriae; elytral interstriae ridged	***C.caudatum***
–	Elytral interstriae with a single row of erect bristles, each longer than width of interstriae; elytral interstriae flat	***C.pygmaeum***
16	Elytral setae very long and thin	***C.kivuensis***
–	Elytral setae of erect bristles or spatulate setae	**17**
17	Interstrial setae in multiple confused rows	**18**
–	Interstrial setae mainly in single rows, multiple confused rows may occur on interstriae 8–10	**21**
18	Frons coarsely granulated; a single (fused) pronotal tooth near anterior margin; elytra apex narrowly rounded	***C.spathifer***
–	Frons almost smooth; two teeth at pronotal margin separated; elytral apex emarginated	**19**
19	Anterior margin of pronotum with pair of bidentate teeth, appearing four-toothed; right elytron just behind scutellar shield with a sharp loop (Madagascar)	***C.quadrispinum* sp. nov.**
–	Anterior margin of pronotum with two uniform teeth; area near scutellar shield only slightly curved	**20**
20	Colour dark brown or black; most elytral setae narrow and bristle-like, shorter than width of an interstria; strial punctures separated by 2–3× their diameter	***C.atrum***
–	Colour brown to black, setae on elytra variegated, scale-like, longest setae as long or longer than width of an interstriae; strial punctures closely set, separated by less than their diameter	***C.methneri***
21	Setae laterally on the metaventrite hair-like; lower elytral declivity in lateral view curved with apex extended slightly posteriorly	***C.acuminatum***
–	Setae laterally on the metaventrite short and plumose on anterior half, with increasingly long scales posteriorly near margin for reception of metafemur; lower declivity in lateral view more or less straight	**22**
22	Elytral interstriae with scattered setae placed in single rows, each seta as long or longer than width of one interstriae	***C.amanicum***
–	Interstriae 810 often with multiple confused rows of densely placed setae, all interstrial setae shorter than width of an interstria, separated within rows by less than their length	**23**
23	Elytral interstriae with the majority of setae spatulate, curved or semirecumbent, setae on declivital interstriae 1 and 2 obliquely directed sideways; interstrial punctures scattered, shallow (South Africa)	***C.uniseriatum***
–	All elytral setae bristle-like, erect; interstrial punctures distinct, densely placed (tropical Africa, Madagascar)	***C.spinifer***

## Supplementary Material

XML Treatment for Ctonoxylon

XML Treatment for Ctonoxylon hirtellum

XML Treatment for Ctonoxylon festivum

XML Treatment for Ctonoxylon flavescens

XML Treatment for Ctonoxylon bosqueiae

XML Treatment for Ctonoxylon hirsutum

XML Treatment for Ctonoxylon tuberculatum

XML Treatment for Ctonoxylon montanum

XML Treatment for Ctonoxylon camerunum

XML Treatment for Ctonoxylon cornutum

XML Treatment for Ctonoxylon torquatum

XML Treatment for Ctonoxylon auratum

XML Treatment for Ctonoxylon pilosum

XML Treatment for Ctonoxylon pygmaeum

XML Treatment for Ctonoxylon caudatum

XML Treatment for Ctonoxylon crenatum

XML Treatment for Ctonoxylon kivuensis

XML Treatment for Ctonoxylon quadrispinum

XML Treatment for Ctonoxylon methneri

XML Treatment for Ctonoxylon atrum

XML Treatment for Ctonoxylon acuminatum

XML Treatment for Ctonoxylon spathifer

XML Treatment for Ctonoxylon amanicum

XML Treatment for Ctonoxylon uniseriatum

XML Treatment for Ctonoxylon spinifer

## ﻿Additional information

### Author ORCIDs

Bjarte H. Jordal https://orcid.org/0000-0001-6082-443X

### Data availability

All of the data that support the findings of this study are available in the main text.
